# New Octadecanoid Enantiomers from the Whole Plants of *Plantago depressa*

**DOI:** 10.3390/molecules23071723

**Published:** 2018-07-14

**Authors:** Xiu-Qing Song, Kongkai Zhu, Jin-Hai Yu, Qianqian Zhang, Yuying Zhang, Fei He, Zhi-Qiang Cheng, Cheng-Shi Jiang, Jie Bao, Hua Zhang

**Affiliations:** 1 School of Chemistry and Chemical Engineering, University of Jinan, 336 West Road of Nan Xinzhuang, Jinan 250022, China; 13156019194@163.com; 2 School of Biological Science and Technology, University of Jinan, 336 West Road of Nan Xinzhuang, Jinan 250022, China; hkhhh.k@163.com (K.Z.); yujinhai12@sina.com (J.-H.Y.); zhangqian464x@163.com (Q.Z.); yuyingzhang2008@163.com (Y.Z.); 18864838287@163.com (F.H.); czq13515312897@163.com (Z.-Q.C.); jiangchengshi-20@163.com (C.-S.J.)

**Keywords:** *Plantago depressa*, octadecanoid, fatty acid, natural enantiomer, anti-inflammation

## Abstract

In this study, 19 octadecanoid derivatives—four pairs of enantiomers (**1**–**8**), two racemic/scalemic mixtures (**9**–**10**), and nine biosynthetically related analogues—were obtained from the ethanolic extract of a Chinese medicinal plant, *Plantago depressa* Willd. Their structures were elucidated on the basis of detailed spectroscopic analyses, with the absolute configurations of the new compounds assigned by time-dependent density functional theory (TD-DFT)-based electronic circular dichroism (ECD) calculations. Six of them (**1**, **3**–**6,** and **9**) were reported for the first time, while **2**, **7,** and **8** have been previously described as derivatives and are currently obtained as natural products. Our bioassays have established that selective compounds show in vitro anti-inflammatory activity by inhibiting lipopolysaccharide-induced nitric oxide (NO) production in mouse macrophage RAW 264.7 cells.

## 1. Introduction

**Figure 1:**
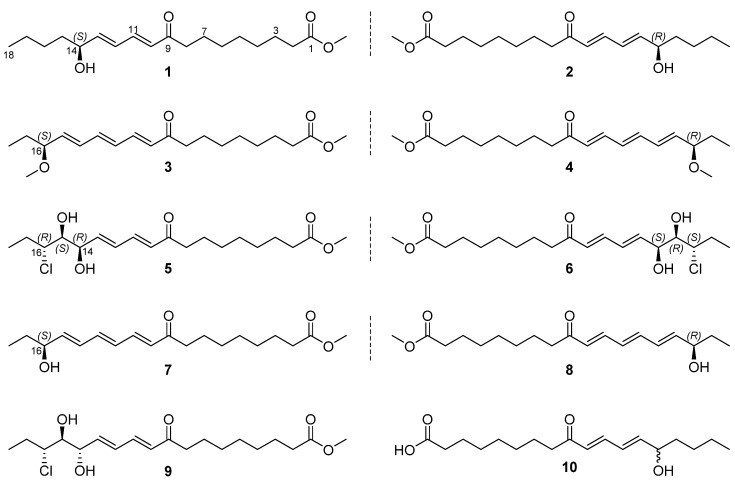
Chemical structures of **1**–**10** from *Plantago depressa.*

The genus *Plantago* L. (family Plantaginaceae) consists of more than 190 species that are widely distributed in temperate and tropical areas all over the world. There are 20 *Plantago* plants that grow in China, including two invasive and one cultivated species [1]. *P. depressa* Willd. is a very common species found in most Asian countries [1], and its whole plants have long been used in traditional Chinese medicine as “Cheqian Cao” for the treatment of oedema, cough, carbuncle, etc. [2]. Previous chemical investigations of this medicinal plant have revealed the presence of phenylethanoid glycosides [3,4,5], iridoid glucosides [6,7], alkaloids [8], and so on [6,7,9]. However, few reports have dealt with the lipid constituents from *P. depressa* until now [10]. In the present work, we carried out an intensive chemical study on the EtOAc partition generated from the ethanolic extract of the whole plants of *P. depressa*, which resulted in the isolation of a series of fatty acid derivatives—four pairs of enantiomers (**1**–**8**), two racemic/scalemic mixtures (**9**–**10**) (Figure 1), and nine related analogues (**11**–**19**). The structures of these compounds were fully characterized by comprehensive spectroscopic analyses, with the absolute stereochemistry of the new compounds established via calculated ECD data. The in vitro antimicrobial, anti-acetylcholinesterase, and anti-inflammatory activities of these lipid molecules were tested; only two known compounds exhibited moderate anti-inflammatory effects. Herein, we describe the separation, structural characterization, and biological evaluations of these plant lipids.

## 2. Results

**Figure 2:**
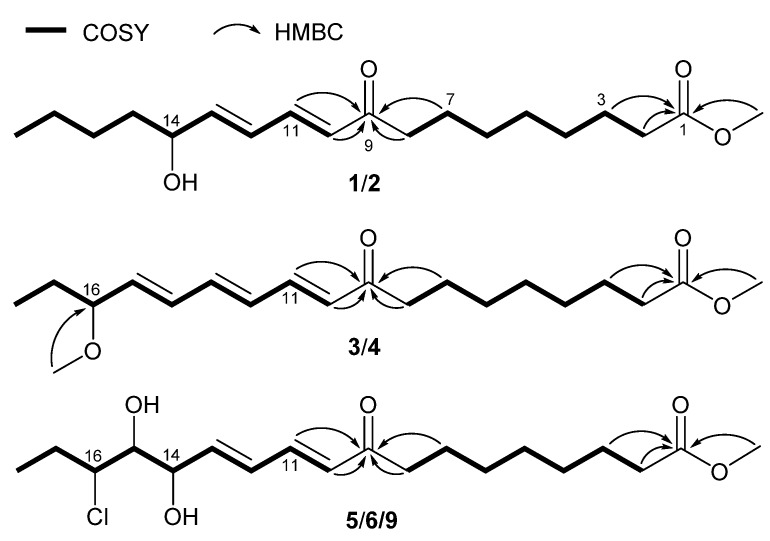
^1^H-^1^H COSY and selected HMBC correlations for **1**–**6** and **9**.

**Figure 3:**
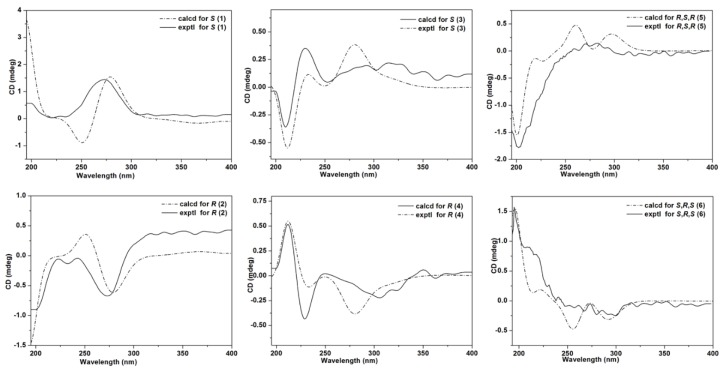
Experimental and calculated ECD spectra for **1**–**6**.

Compounds **1**/**2**—colorless gum—were assigned the molecular formula of C_19_H_32_O_4_ by positive mode high resolution electrospray ionization mass spectrometry [(+)-HR-ESIMS] analysis at *m*/*z* 325.2368 ([M + H]^+^, calcd 325.2373). The ^1^H NMR (nuclear magnetic resonance) data (Table 1) revealed the presence of a conjugated diene [6.20 (d, *J* = 15.6 Hz, H-10), 6.25 (dd, *J* = 15.3, 5.9 Hz, H-13), 6.41 (dd, *J* = 15.3, 10.8 Hz, H-12), 7.27 (dd, *J* = 15.6, 10.8 Hz, H-11)], an oxygenated methine (*δ*_H_ 4.17, m), a methoxy (*δ*_H_ 3.65, s), and a methyl [*δ*_H_ 0.92 (t, *J* = 7.4 Hz)] group. The ^13^C NMR data (Table 2) showed signals of a conjugated ketone (*δ*_C_ 203.7, C-9), an ester carbonyl (*δ*_C_ 176.0, C-1), four olefinic (*δ*_C_ 128.8, 130.3, 144.3, 148.5; C-10 to C-13), an oxygenated methine (*δ*_C_ 72.6, C-14), a methoxy (*δ*_C_ 52.0), ten aliphatic methylene (*δ*_C_ 23.7, 25.5, 26.0, 28.7, 30.0, 30.1, 30.2, 37.8, 34.8, 41.0), and a methyl (*δ*_C_ 14.4, C-18) carbon. These spectral features were similar to those of compound **10** isolated from the fungus *Pleurocybella porrigens* [11] but with an additional methoxy group, suggestive of a methyl ester derivative. Detailed examination of 2D ^1^H-^1^H COSY (correlated spectroscopy) and HMBC (heteronuclear multiple-bond correlation) data (Figure 2) confirmed the above conclusion with key HMBC correlations from H_2_-7, H_2_-8, H-10 and H-11 to C-9 (*δ*_C_ 203.7), and H_2_-2, H_2_-3 and OC*H*_3_ to C-1 (*δ*_C_ 176.0,). Therefore, compounds **1**/**2** were characterized as methyl (10*E*,12*E*)-14-hydroxy-9-oxo-10,12-octadecadienoate. Further spectroscopic analyses revealed that compared with the reported (–)-enantiomer of compound **10** (porrigenic acid) [12], **1**/**2** were neither active enough in the [*α*]_D_ measurement, nor showed decent Cotton effect in the ECD experiment; this alerted us of its racemic or scalemic nature. Subsequent chiral high performance liquid chromatography (HPLC) analysis clearly revealed a pair of enantiomers in a ratio of *ca.* 55:45 (Supplementary Information Figure S2). On reviewing the literature, the (+)-enantiomer (**1**) was identified to be a new compound, while the (−)-enantiomer (**2**) was a new natural product that had been reported as the methyl ester of porrigenic acid (**10**) during structure characterization [12]. It is worth noting that the absolute configuration of compound **2** was initially determined as *S* using allylic benzoate method [12]. However, the assignment was apparently not rigorous because this ECD method was originally developed to assign absolute stereochemistry of allylic alcohol (hydroxyl group adjacent to a double bond chromophore) [13], but the chromophore in compound **2** is an α,β,γ,δ-conjugated ketone. We therefore employed the time-dependent density functional theory (TD-DFT) method to calculate the ECD spectra (Figure 3) of the two enantiomers and finally differentiated them from each other.

Compounds **3**/**4** had the molecular formula of C_20_H_32_O_4_ as deduced from the (+)-HR-ESIMS ion peak at *m*/*z* 337.2369 ([M + H]^+^, calcd 337.2373), which was 14 mass units (CH_2_) more than that of compounds **7**/**8** [14,15] indicative of a methylated analogue. Analysis of the NMR data (Table 1 and Table 2) for compounds **3**/**4** confirmed this hypothesis, with extra signals for a methoxy group (*δ*_H_ 3.28, *δ*_C_ 56.7) and the downfield shifted C-16 resonance (*δ*_C_ 84.5) in contrast with that (*δ*_C_ 74.2) in compounds **7**/**8**. Further inspection of 2D NMR data (Figure 2) corroborated this structural assignment, revealing key HMBC correlations with the methoxy protons to C-16. Compounds **3**/**4** were thus characterized to be methyl (10*E*,12*E*,14*E*)-16-methoxy-9-oxo-10,12,14-octadecatrienoate. Similar to compounds **1**/**2**, the optical rotation and ECD data of compounds **3**/**4** suggested a scalemic mixture with nearly zero [*α*]_D_ and no Cotton effect, respectively. The two pure enantiomers were further separated from each other by chiral HPLC and structurally differentiated by comparing their experimental ECD spectra with the calculated ones (Figure 3).

Compounds **5**/**6** were determined to be monochlorinated on the basis of the ESIMS (electrospray ionization mass spectrometry) isotope ion peak at *m*/*z* 397.1/399.1 ([M + Na]^+^, ca. 3:1) and were assigned the molecular formula of C_19_H_32_O_5_Cl by (+)-HR-ESIMS analysis at *m*/*z* 397.1756 ([M + Na]^+^, calcd 397.1752). The NMR data (Table 1 and Table 2) for compounds **5**/**6** also displayed resonances for several functional groups as those in compounds **1**/**2**, such as two carbonyls (*δ*_C_ 174.5 and 201.0), a diene (*δ*_C_ 130.6, 141.1 and 130.3, 141.7; *δ*_H_ 6.20, 7.15 and 6.49, 6.21), and an ester methoxyl (*δ*_C_ 51.7; *δ*_H_ 3.67). Meanwhile, compounds **5**/**6** possessed three sp^3^ methines (*δ*_C_ 64.6, 71.0 and 76.5; *δ*_H_ 3.97, 4.70 and 3.62) compared with only one in compounds **1**/**2**, which was ascribed to two hydroxyl and a chlorine substituents by analyzing the molecular composition and chemical shifts of these methines. Subsequent acquisition of 2D ^1^H-^1^H COSY and HMBC data (Figure 2) confirmed the establishment of the planar structure of compounds **5**/**6** as shown, and the substitution pattern of 14-OH, 15-OH, and 16-Cl for the C-14–C-16 fragment was supported by the lower chemical shift for C-16 than those for C-14 and C-15 [16]. The relative configuration of compounds **5**/**6** was determined by the *J*-based configuration analysis method [17]. The magnitudes of *J*_14,15_ (2.7 Hz) and *J*_15,16_ (7.6 Hz) indicated a *syn*-relationship between H-14 and H-15 and an *anti*-relationship between H-15 and H-16, respectively. Alerted by the cases of compounds **1**–**4**, compounds **5**/**6** were also subjected to chiral HPLC analysis and indeed proved to be another pair of enantiomers. The absolute configurations of compounds **5**/**6** were further assigned by comparing their experimental ECD spectra with the computed ones (Figure 3).

Compound **9** was assigned the molecular formula of C_19_H_32_O_5_Cl—same as compounds **5**/**6**—based on the (+)-HR-ESIMS ion peak at *m*/*z* 397.1757 ([M + Na]^+^, calcd 397.1752), supportive of an isomer of the latter. Analysis of the NMR data (Table 1 and Table 2) for compound **9** corroborated this conclusion, with very similar NMR data suggesting that they were diastereoisomers of the same planar structure; this was further confirmed by examination of 2D NMR correlations (Figure 2). Detailed NMR comparison between compounds **9** and **5**/**6** revealed nearly superimposable ^1^H and ^13^C NMR spectra, and the only difference was attributable to signals across CH-13 to CH-16 moiety. Obvious NMR variations were observed for resonances from H-13 to H-16, C-13 to C-14, and *J*_14,15_ (Table 1 and Table 2), which all suggested an inverted C-14 configuration in compound **9** compared with that in compounds **5**/**6**. The structure and relative configuration of compound **9** were thus elucidated. It was inferable that compound **9** could also be enantiomeric mixture in light of the aforementioned examples of its cometabolites. However, it was not further separated on chiral HPLC due to degradation during storage, as indicated by subsequent ^1^H NMR measurement. Moreover, the scarce amount of sample prevented us from further investigation.

In addition to the above-described molecules, compounds **7**/**8** were also demonstrated to be enantiomeric mixtures and separated by chiral HPLC. They had been reported in mixture as the methylation derivatives of their corresponding fatty acids [14,15], and we herein report them as enantiomerically pure isolates as new natural products. Compound **10** had been previously obtained in scalemic form [11] and (−)-form [12], respectively, from the same fungus by two Japanese research groups. In the current work, it was obtained as a nearly racemic mixture (${\left[\alpha \right]}_{D}^{21}$ 0.3; *c* 0.10, MeOH) and was not separable on both normal-phase and reversed-phase chiral HPLC columns. The other known analogues were identified to be (9*Z*,12*Z*,14*E*)-16-oxo-octadecatrienoic acid (**11**) [18], (10*Z*,12*E*,14*Z*)-9,16-dioxo-octadecatrienoic acid (**12**) [19], linoleic acid (**13**) [20], *β*-(9′*Z*,12′*Z*,15′*Z*)-octadecatrienoic acid monoglyceride (**14**) [21], 1-*O*-(9Z,12Z)-octadecadienoyl glycerol (**15**) [22], *α*-(9′*Z*,12′*Z*,15′*Z*)-octadecatrienoic acid monoglyceride (**16**) [21], 1-*O*-(10*E*,12*E*)-9-oxo-octadecadienoyl glycerol (**17**) [23], 1-*O*-(9*Z*,11*E*)-13-oxo-octadecadienoyl glycerol (**18**) [24], and 1-*O*-(9*Z*,11*E*)-9-oxo-octadecadienoyl glycerol (**19**) [24] by spectroscopic data.

Most compounds (only those with enough amount) were screened for their antimicrobial, anti-acetylcholinesterase, and anti-inflammatory activities (Supplementary Information Tables S1 and S2); only compounds **13** and **18** displayed anti-inflammatory effect with moderate inhibition against nitric oxide (NO) production with IC_50_ values of 13.08 ± 0.25 and 7.64 ± 0.21 μM, respectively.

## 3. Materials and Methods

### 3.1. General Experimental Procedures

Optical rotations were measured on a Rudolph VI polarimeter (Rudolph Research Analytical, Hackettstown, NJ, USA) with a 10 cm length cell. NMR experiments were recorded on a Bruker Avance DRX600 spectrometer (Bruker BioSpin AG, Fallanden, Switzerland) and referenced to residual solvent peaks (CD_3_OD: *δ*_H_ 3.31, *δ*_C_ 49.00; CDCl_3_: *δ*_H_ 7.26, *δ*_C_ 77.16). HR-ESIMS spectra were obtained on an Agilent 6545 Q-TOF mass spectrometer (Agilent Technologies Inc., Waldbronn, Germany). ESIMS analyses were carried out on an Agilent 1260-6460 Triple Quad LC-MS instrument (Agilent Technologies Inc., Waldbronn, Germany). UV spectra were obtained on a Shimadzu UV-2600 spectrophotometer (Shimadzu, Kyoto, Japan) with a 1 cm pathway cell. Normal HPLC separation was performed using an Agilent 1260 series LC instruments (Agilent Technologies Inc., Waldbronn, Germany) coupled with an Agilent SB-C_18_ (9.4 × 250 mm) column (Agilent Technologies Inc., Santa Clara, CA, USA). Chiral MZ(2) RH 5u (4.6 × 250 mm) chiral column (Phenomenex, Washington, CD, USA) and CHIRALPAK AD-H (4.6 × 250 mm) chiral column (Daicel Corporation, Tokyo, Japan) were used for chiral HPLC analysis and separation. ECD spectra were acquired on a Chirascan circular dichroism spectrometer (Applied Photophysics Ltd., Surrey, UK). Column chromatography (CC) was performed on D101-macroporous absorption resin (Sinopharm Chemical Reagent Co., Ltd., Shanghai, China), MCI gel (CHP20P, Mitsubishi Chemical Corporation, Tokyo, Japan), reversed phase C18 silica gel (Merck KGaA, Darmstadt, Germany), Sephadex LH-20 (GE Healthcare Bio-Sciences AB, Uppsala, Sweden), and silica gel (300–400 mesh; Qingdao Marine Chemical Ltd., Qingdao, China). All solvents used for CC were of analytical grade (Tianjin Fuyu Fine Chemical Co., Ltd., Tianjin, China) and solvents used for HPLC were of HPLC grade (Oceanpak Alexative Chemical Ltd., Goteborg, Sweden). Pre-coated silica gel GF254 plates (Qingdao Haiyang Chemical Co., Ltd., Qingdao, China) were used for thin-layer chromatography (TLC) monitoring.

### 3.2. Plant Material

The whole plants of *P. depressa* were collected in June 2016 at Mount Kunyu, Shandong Province, and were authenticated by Prof. Jie Zhou from University of Jinan. A voucher specimen has been deposited at School of Biological Science and Technology, University of Jinan (Accession number: npmc-007).

### 3.3. Extraction and Isolation

The air-dried powder of the whole plants of *P. depressa* (15 kg) was extracted with 95% EtOH at room temperature three times to afford a crude extract (0.9 kg). The extract was then suspended in 2.0 L water and partitioned with EtOAc (2.0 L × 3). The EtOAc extract (300 g) was subjected to CC over D101-macroporous absorption resin and eluted with EtOH-H_2_O (30%, 50%, 80% and 95%) to afford four fractions (A–D). Fraction C (80%, 87 g) was subjected to passage over an MCI gel column and eluted with MeOH-H_2_O (50% to 100%) to give five subfractions (C1–C5). C1 was then separated by silica gel CC eluted with petroleum ether-acetone (4:1 to 1:1) to produce two eluents (C1-1 and C1-2), and C1-1 was further purified by HPLC (3.0 mL/min 80% MeOH-H_2_O, *t*_R_ = 10.0 min) to afford **7**/**8** (3.1 mg). Fraction C2 was then separated by silica gel CC eluted with petroleum ether-acetone (8:1 to 1:1) to produce eleven subfractions (C2-1–C2-11). C2-4 was subjected to silica gel CC eluted with CHCl_3_-MeOH (100:1 to 10:1) to give five major eluents (C2-4-1–C2-4-5), and C2-4-4 was then purified by HPLC (3.0 mL/min 80% MeOH-H_2_O, *t*_R_ = 15.0 min) to afford **3**/**4** (1.0 mg). Fraction C2-6 was subjected to silica gel CC eluted with CHCl_3_-MeOH (100:1 to 10:1) to give two major subfractions (C2-6-1 and C2-6-2), C2-6-2 was then purified by HPLC (3.0 mL/min 80% MeOH-H_2_O, *t*_R_ = 9.5 min) to afford **1**/**2** (3.5 mg). Fraction C2-11 was chromatographed on an RP-C18 silica gel column to give three subfractions (C2-11-1–C2-11-3), and C2-11-3 was then purified by HPLC (3.0 mL/min 80% MeOH-H_2_O, *t*_R_ = 10.0 min and 11.0 min, respectively) to afford **9** (2.5 mg) and **5**/**6** (2.0 mg). Fraction C3 was then separated by silica gel CC eluted with petroleum ether-acetone (20:1 to 1:1) to produce five subfractions (C3-1–C3-5), and C3-4 was then chromatographed on an RP-C18 silica gel column to give seven eluents (C3-4-1–C3-4-7). Fraction C3-4-7 was subjected to silica gel CC eluted with CHCl_3_-MeOH (200:1 to 10:1) to give two major fractions—C3-4-7-1 and C3-4-7-2—and C3-4-7-1 was further purified by HPLC (3.0 mL/min 90% MeOH-H_2_O, *t*_R_ = 15.5 min) to afford **12** (5.0 mg). Fraction C3-5 was chromatographed on an RP-C18 silica gel column to give seven subfractions (C3-5-1–C3-5-7). Fraction C3-5-3 was subjected to Sephadex LH-20 CC to give two subfractions (C3-5-3-1–C3-5-3-2), and C3-5-3-1 was further purified by HPLC (3.0 mL/min 90% MeOH-H_2_O, *t*_R_ = 11.0 min) to afford **10** (1.0 mg). Fraction C3-5-7 was subjected to Sephadex LH-20 CC to give one major fraction (C3-5-7-1), which was further purified by HPLC (3.0 mL/min 95% MeOH-H_2_O, *t*_R_ = 10.0 min, 11.0 min and 12.5 min, respectively) to afford **17** (1.0 mg), **18** (1.2 mg), and **19** (1.0 mg). Fraction C4 was then separated by silica gel CC eluted with petroleum ether-acetone (20:1 to 1:1) to produce six subfractions (C4-1–C4-6). Fraction C4-1 was chromatographed on an RP-C18 silica gel column to give twelve subfractions (C4-1-1–C4-1-12), and C4-1-12 was then purified by HPLC (3.0 mL/min 85% MeOH-H_2_O, *t*_R_ = 7.5 min and 10.0 min, respectively) to afford **15** (2.8 mg) and **16** (1.6 mg). Fraction C4-4 was chromatographed on an RP-C18 silica gel column to give four subfractions (C4-4-1–C4-4-4). Fraction C4-4-4 was subjected to silica gel CC eluted with CHCl_3_-MeOH (200:1 to 10:1) to give five major eluents (C4-4-4-1–C4-4-4-5), and C4-4-4-5 was further purified by HPLC (3.0 mL/min 85% MeOH-H_2_O, *t*_R_ = 10.0 min) to afford **11** (1.8 mg). Fraction C4-6 was subjected to Sephadex LH-20 CC to give six subfractions (C4-6-1–C4-6-6). C4-6-6 was subjected to silica gel CC eluted with petroleum ether-acetone (50:1 to 1:1) to give two major eluents (C4-6-6-1–C4-6-6-2), and C4-6-6-2 was then purified by HPLC (3.0 mL/min 90% MeOH-H_2_O, *t*_R_ = 11.0 min and 17.5 min, respectively) to afford **14** (5 mg) and **13** (1 mg).

Furthermore, compounds **1**–**8** were separated by chiral HPLC on a Chiral MZ(2) RH column as follows: 1.0 mL/min MeCN to yield **2** (0.7 mg, *t*_R_ = 1.8 min) and **1** (1.2 mg, *t*_R_ = 2.4 min), 1.0 mL/min 80% MeCN-H_2_O to afford **3** (0.2 mg, *t*_R_ = 11.6 min) and **4** (0.2 mg, *t*_R_ = 12.4 min), 1.0 mL/min 80% MeCN-H_2_O to give **6** (0.3 mg, *t*_R_ = 4.5 min) and **5** (0.2 mg, *t*_R_ = 4.9 min), and 1.0 mL/min 80% MeCN-H_2_O to furnish **7** (1.1 mg, *t*_R_ = 6.8 min) and **8** (1.0 mg, *t*_R_ = 7.3 min).

Compounds **1**/**2**: Colorless gum; ${\left[\alpha \right]}_{D}^{21}$ +29.1 (**1**: *c* 0.12, MeOH) and −27.1 (**2**: *c* 0.07, MeOH); UV (MeOH) *λ*_max_ (log *ε*) 275 (3.2); ^1^H NMR (CD_3_OD) see Table 1; ^13^C NMR (CD_3_OD) see Table 2; (+)-ESIMS *m*/*z* 347.1 [M + Na]^+^; (+)-HR-ESIMS *m*/*z* 325.2368 [M + H]^+^ (calcd for C_19_H_33_O_4_, 325.2373).

Compounds **3**/**4**: Colorless gum; ${\left[\alpha \right]}_{D}^{21}$ −10.0 (**3**: *c* 0.01, MeOH) and +11.1 (**4**: *c* 0.01, MeOH); UV (MeOH) *λ*_max_ (log *ε*) 310 (3.4); ^1^H NMR (CD_3_OD) see Table 1; ^13^C NMR (CD_3_OD) see Table 2; (+)-ESIMS *m*/*z* 359.2 [M + Na]^+^; (+)-HR-ESIMS *m*/*z* 337.2369 [M + H]^+^ (calcd for C_20_H_33_O_4_, 337.2373).

Compounds **5**/**6**: Colorless gum; ${\left[\alpha \right]}_{D}^{21}$ −12.6 (**5**: *c* 0.02, MeOH) and +14.1 (**6**: *c* 0.04, MeOH); UV (MeOH) *λ*_max_ (log *ε*) 265 (3.5); ^1^H NMR (CDCl_3_) see Table 1; ^13^C NMR (CDCl_3_) see Table 2; (+)-ESIMS *m*/*z* 397.1 [M + Na]^+^; (+)-HR-ESIMS *m*/*z* 397.1756 [M + Na]^+^ (calcd for C_19_H_32_O_5_Cl, 397.1752).

Compound **9**: Colorless gum; ^1^H NMR (CDCl_3_) see Table 1; ^13^C NMR (CDCl_3_) see Table 2; (+)-ESIMS *m*/*z* 397.1 [M + Na]^+^; (+)-HR-ESIMS *m*/*z* 397.1757 [M + Na]^+^ (calcd for C_19_H_32_O_5_Cl, 397.1752).

### 3.4. Antimicrobial Assay

The antimicrobial assays were performed as we have reported earlier [25].

### 3.5. Anti-Acetylcholinesterase Assay

The anti-acetylcholinesterase assay was conducted as we have described earlier [26].

### 3.6. Anti-Inflammatory Assay

Determination of nitric oxide production. Briefly, RAW 264.7 cells were plated into 96-well plates and pretreated with a series of concentrations of compounds for 1 h before treatment with 1 μg/mL LPS. After 24 h incubation, detection of accumulated nitric oxide in the cell supernatants was assayed by Griess reagent kit (Beyotime Institute of Biotechnology) according to the manufacturer’s instructions. Equal volumes of culture supernatant and Griess reagent were mixed, and the absorbance at 540 nm was measured using a Microplate Reader (Tecan, Switzerland).

Cell viability assay. RAW 264.7 cells were seeded into 96-well plates at 1 × 10^4^ cells/well and allowed to attach for 24 h. The medium was replaced with 100 μL medium containing the indicated concentrations of compounds and further incubated for 24 h. 10 μL of MTT (5 mg/mL in PBS) was added into each well and the plates were incubated for 4 h at 37 °C. Supernatants were aspirated and formed formazan was dissolved in 100 μL of dimethyl sulfoxide (DMSO). The optical density (OD) was measured at an absorbance wavelength of 490 nm using a Microplate Reader (Tecan, Switzerland).

### 3.7. ECD Calculations

Conformational analysis within an energy window of 3.0 kcal/mol was performed by using the OPLS3 [27,28] molecular mechanics force field via the MacroModel [29] panel of Maestro 10.2. The conformers were then further optimized with the software package Gaussian 09 [30] at the B3LYP/6-311++G(2d,*p*) level, and the harmonic vibrational frequencies were also calculated to confirm their stability. Then, the 30 lowest electronic transitions for the obtained conformers in vacuum were calculated using TD-DFT method at the B3LYP/6-311++G(2d,*p*) level. ECD spectra of the conformers were simulated using a Gaussian function with a half-bandwidth of 0.26 eV. The overall theoretical ECD spectra were obtained according to the Boltzmann weighting of each conformer.

## Figures and Tables

**Table 1:** ^1^H NMR data for **1**–**6** and **9** (600 MHz).

Position	1/2 ^a^	3/4 ^a^	5/6 ^b^	9 ^b^
2	2.32, t (7.4)	2.31, t (7.4)	2.29, t (7.6)	2.30, t (7.5)
3	1.60, m	1.61, m	1.61, m	1.62, m
4	1.34, m	1.33, m	1.32, m	1.32, m
5	1.34, m	1.33, m	1.32, m	1.32, m
6	1.34, m	1.33, m	1.32, m	1.32, m
7	1.60, m	1.61, m	1.61, m	1.61, m
8	2.61, t (7.3)	2.61, t (7.4)	2.55, t (7.5)	2.55, t (7.4)
10	6.20, d (15.6)	6.23, d (15.5)	6.20, d (15.6)	6.21, d (15.5)
11	7.27, dd (15.6, 10.8)	7.31, dd (15.5, 11.2)	7.15, dd (15.6, 10.8)	7.16, dd (15.6, 11.0)
12	6.41, dd (15.3, 10.8)	6.43, dd (15.1, 11.2)	6.49, dd (15.5, 10.8)	6.50, dd (15.3, 11.0)
13	6.25, dd (15.3, 5.9)	6.75, dd (15.1, 10.9)	6.21, dd (15.5, 6.0)	6.25, dd (15.3, 6.8)
14	4.17, m	6.39, dd (15.2, 10.9)	4.70, dd (6.0, 2.7)	4.62, dd (6.8, 5.1)
15	1.55, m	5.81, dd (15.2, 7.8)	3.62, dd (7.6, 2.7)	3.86, dd (7.7, 5.1)
16	1.34, m	3.62, m	3.97, ddd (9.3, 7.6, 2.9)	3.76, ddd (9.4, 7.7, 2.8)
17	1.37, m	1.61, m	1.76, m	1.74, m
			2.07, m	2.08, m
18	0.92, t (7.4)	0.90, t (7.4)	1.08, t (7.3)	1.07, t (7.2)
1-OMe	3.65, s	3.65, s	3.67, s	3.67, s
16-OMe		3.28, s		

^a^ In CD_3_OD; ^b^ in CDCl_3_.

**Table 2:** ^13^C NMR data for **1**–**6** and **9** (150 MHz).

Position	1/2 ^a^	3/4 ^a^	5/6 ^b^	9 ^b^
1	176.0	176.0	174.5	174.5
2	34.8	34.8	34.2	34.2
3	26.0	26.0	25.0	25.0
4	30.0 ^c^	30.0 ^d^	29.1 ^e^	29.1 ^f^
5	30.1 ^c^	30.2 ^d^	29.2 ^e^	29.2 ^f^
6	30.2 ^c^	30.2 ^d^	29.2 ^e^	29.2 ^f^
7	25.5	25.5	24.3	24.3
8	41.0	41.1	40.8	40.8
9	203.7	203.5	201.0	200.9
10	130.3	130.5	130.6	130.9
11	144.3	144.5	141.1	141.0
12	128.8	131.9	130.3	131.5
13	148.5	142.3	141.7	139.4
14	72.6	133.2	71.0	72.5
15	37.8	140.2	76.5	76.6
16	28.7	84.5	64.6	64.7
17	23.7	29.2	26.8	26.6
18	14.4	9.9	10.7	10.6
1-OMe	52.0	52.0	51.7	51.6
16-OMe		56.7		

^a^ In CD_3_OD; ^b^ in CDCl_3_; ^c–f^ Interchangeable assignments.

## Supplementary Materials

The following materials are available online, raw spectroscopic data including chiral HPLC analyses, HR-ESIMS, and NMR (^1^H, ^13^C, ^1^H-^1^H COSY, HSQC, and HMBC) spectra for new compounds **1**–**6** and **9**.
