# Supplementary material for: New Octadecanoid Enantiomers from the Whole Plants of Plantago depressa
Source: Molecules. 2018 Jul 14;23(7):1723. doi: 10.3390/molecules23071723 (PMC6099667; doi:10.3390/molecules23071723)
Supplement: Supplementary file 1 [file molecules-23-01723-s001.pdf]

Supplementary Information for

New Octadecanoid Enantiomers from the Whole Plants of *Plantago depressa*

Xiu-Qing Song [1,2], Kongkai Zhu [2], Jin-Hai Yu [2], Qianqian Zhang [2], Yuying Zhang[2], Fei He [2], Zhi-Qiang Cheng [2], Cheng-Shi Jiang [2], Jie Bao [2,*], Hua Zhang [2,*]

[1] *School of Chemistry and Chemical Engineering, University of Jinan, 336 West Road of Nan Xinzhuang, Jinan 250022, China*

[2] *School of Biological Science and Technology, University of Jinan, 336 West Road of Nan Xinzhuang, Jinan 250022, China*

Correspondence: bio_baoj@ujn.edu.cn; bio_zhangh@ujn.edu.cn; Tel.: +86-531-89736199

Figure S1. Comparisons of ^{1}H NMR spectra of scalemic mixture and pure enantiomers (**1**–**6**).

Figure S2. Chiral HPLC chromatograms for **1**–**8**.

Figure S3. The ^{1}H NMR spectrum of **1**/**2** in CD_3OD.

Figure S4. The ^{13}C NMR spectrum of **1**/**2** in CD_3OD.

Figure S5. The ^{1}H-^{1}H COSY spectrum of **1**/**2** in CD_3OD.

Figure S6. The HSQC spectrum of **1**/**2** in CD_3OD.

Figure S7. The HMBC spectrum of **1**/**2** in CD_3OD.

Figure S8. The (+)-HRESIMS spectrum of **1**/**2**.

Figure S9. The ^{1}H NMR spectrum of **3**/**4** in CD_3OD.

Figure S10. The ^{13}C NMR spectrum of **3**/**4** in CD_3OD.

Figure S11. The ^{1}H-^{1}H COSY spectrum of **3**/**4** in CD_3OD.

Figure S12. The HSQC spectrum of **3**/**4** in CD_3OD.

Figure S13. The HMBC spectrum of **3**/**4** in CD_3OD.

Figure S14. The (+)-HRESIMS spectrum of **3**/**4**.

Figure S15. The ^{1}H NMR spectrum of **5**/**6** in $CDCl_3$.

Figure S16. The ^{13}C NMR spectrum of **5**/**6** in $CDCl_3$.

Figure S17. The ^{1}H-^{1}H COSY spectrum of **5**/**6** in $CDCl_3$.

Figure S18. The HSQC spectrum of **5**/**6** in $CDCl_3$.

Figure S19. The HMBC spectrum of **5**/**6** in $CDCl_3$.

Figure S20. The (+)-HRESIMS spectrum of **5**/**6**.

Figure S21. The ^{1}H NMR spectrum of **9** in $CDCl_3$.

Figure S22. The ^{13}C NMR spectrum of **9** in $CDCl_3$.

Figure S23. The ^{1}H-^{1}H COSY spectrum of **9** in $CDCl_3$.

Figure S24. The HSQC spectrum of **9** in $CDCl_3$.

Figure S25. The HMBC spectrum of **9** in $CDCl_3$.

Figure S26. The (+)-HRESIMS spectrum of **9**.

Table S1. Preliminary antimicrobial assay results.

Table S2. Preliminary anti-acetylcholinesterase and anti-inflammatory assay results.

Figure S1. Comparisons of ^{1}H NMR spectra of scalemic mixture and pure enantiomers (**1**–**6**).

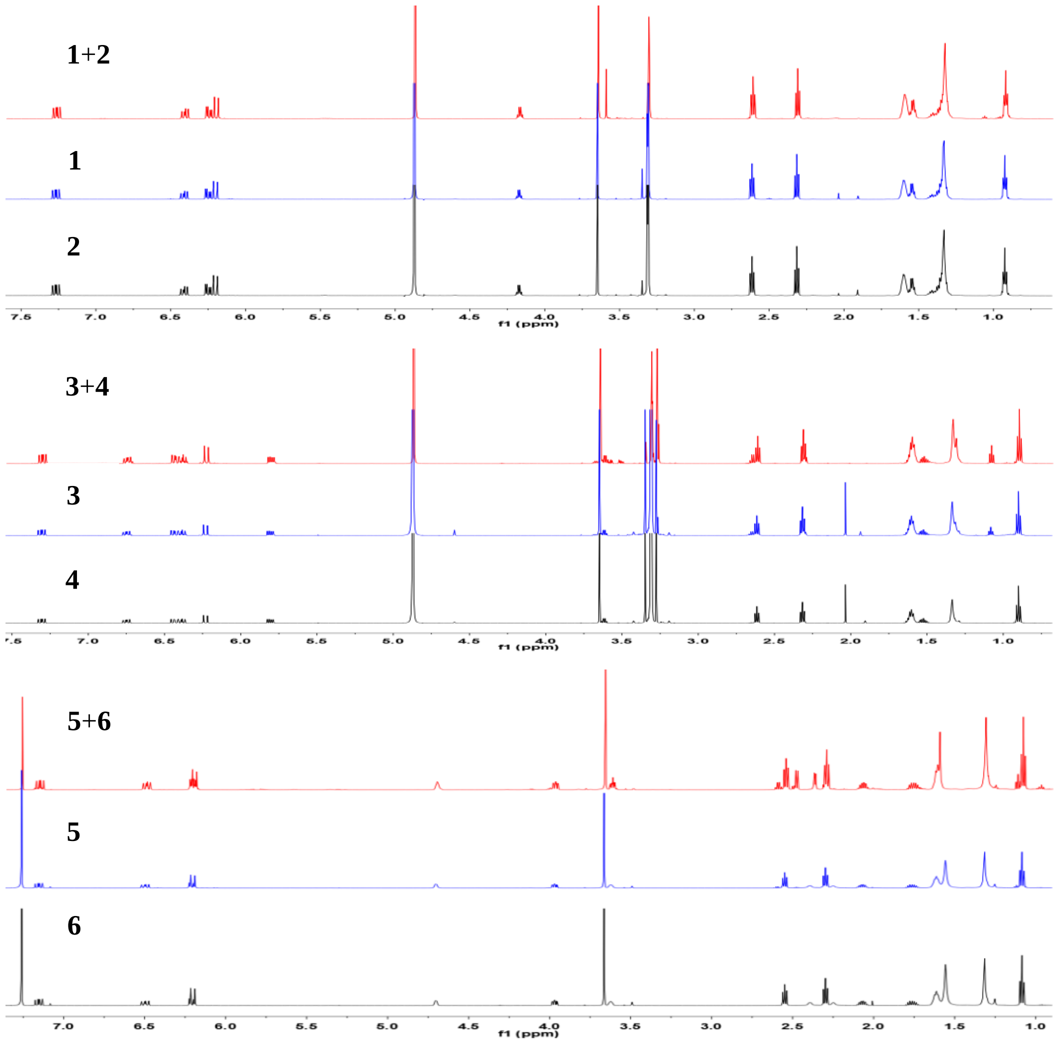

Figure S2. Chiral HPLC chromatograms for **1**–**8**.

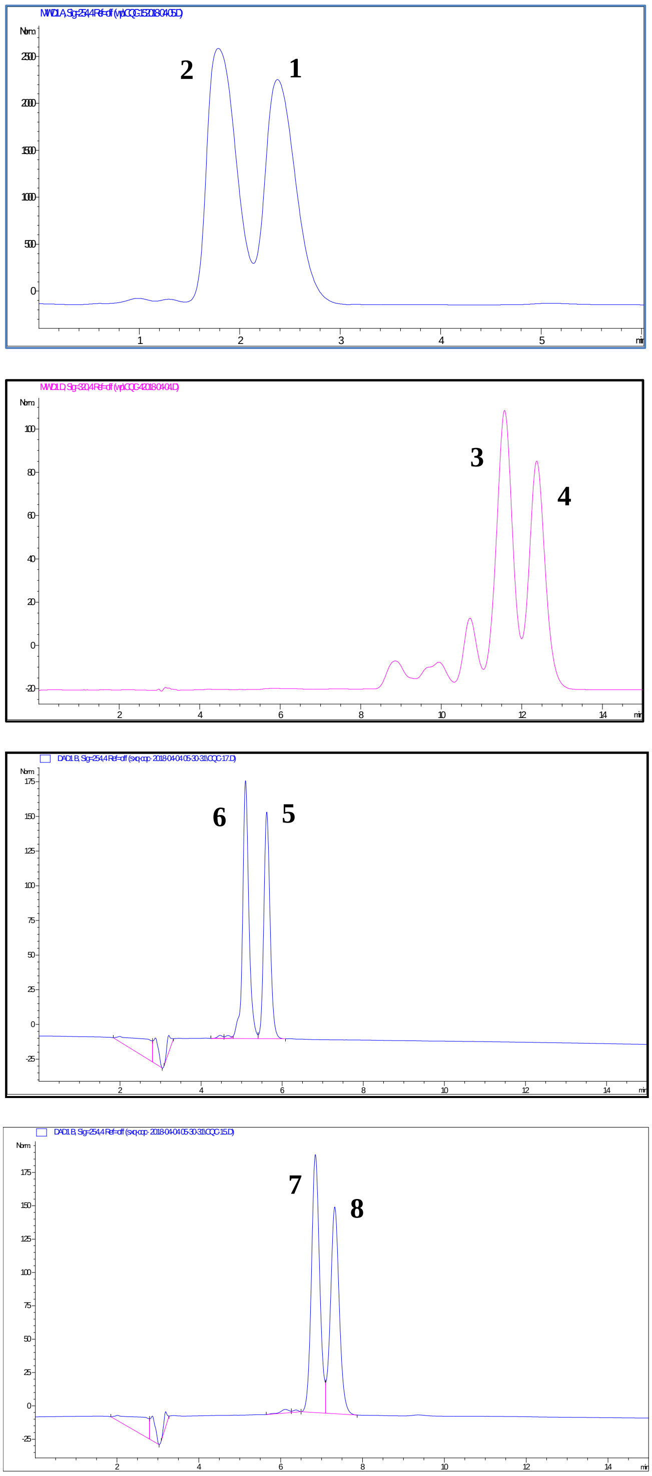

Figure S3. The ^{1}H NMR spectrum of **1**/**2** in CD_3OD

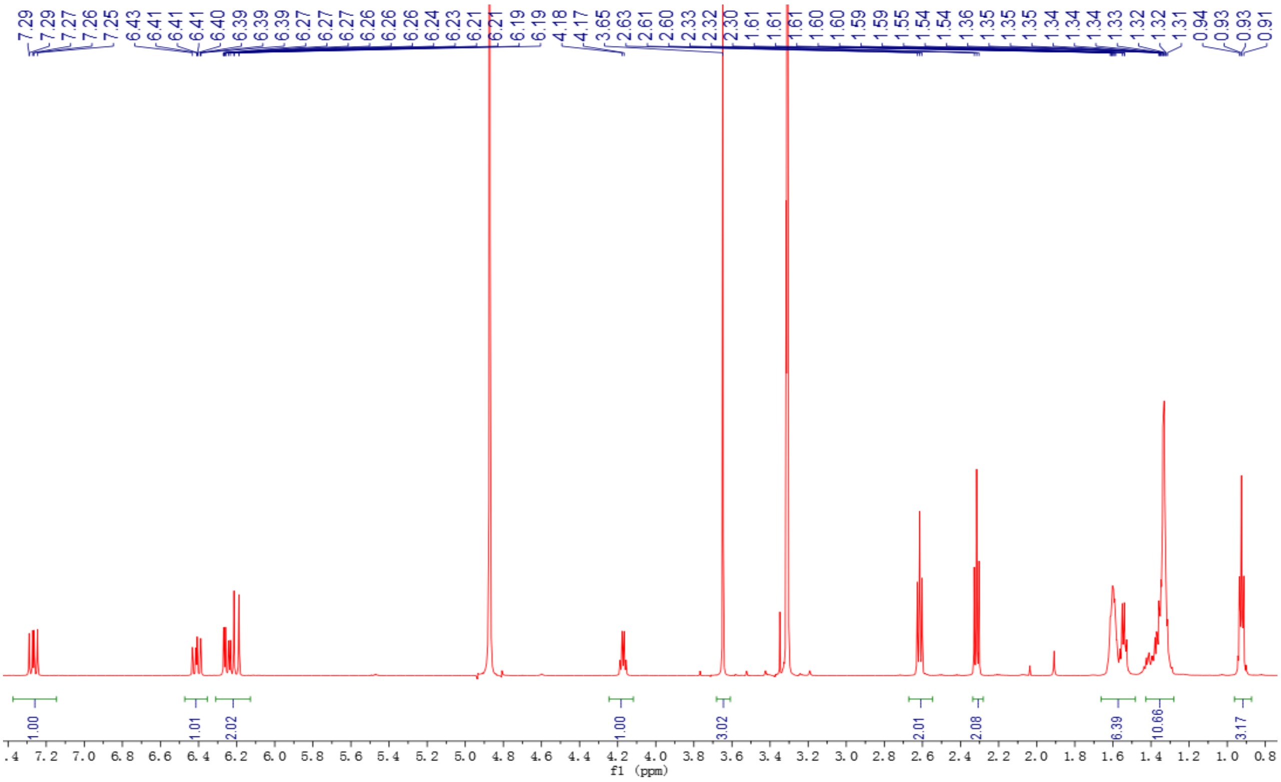

Figure S4. The ^{13}C NMR spectrum of **1**/**2** in CD_3OD

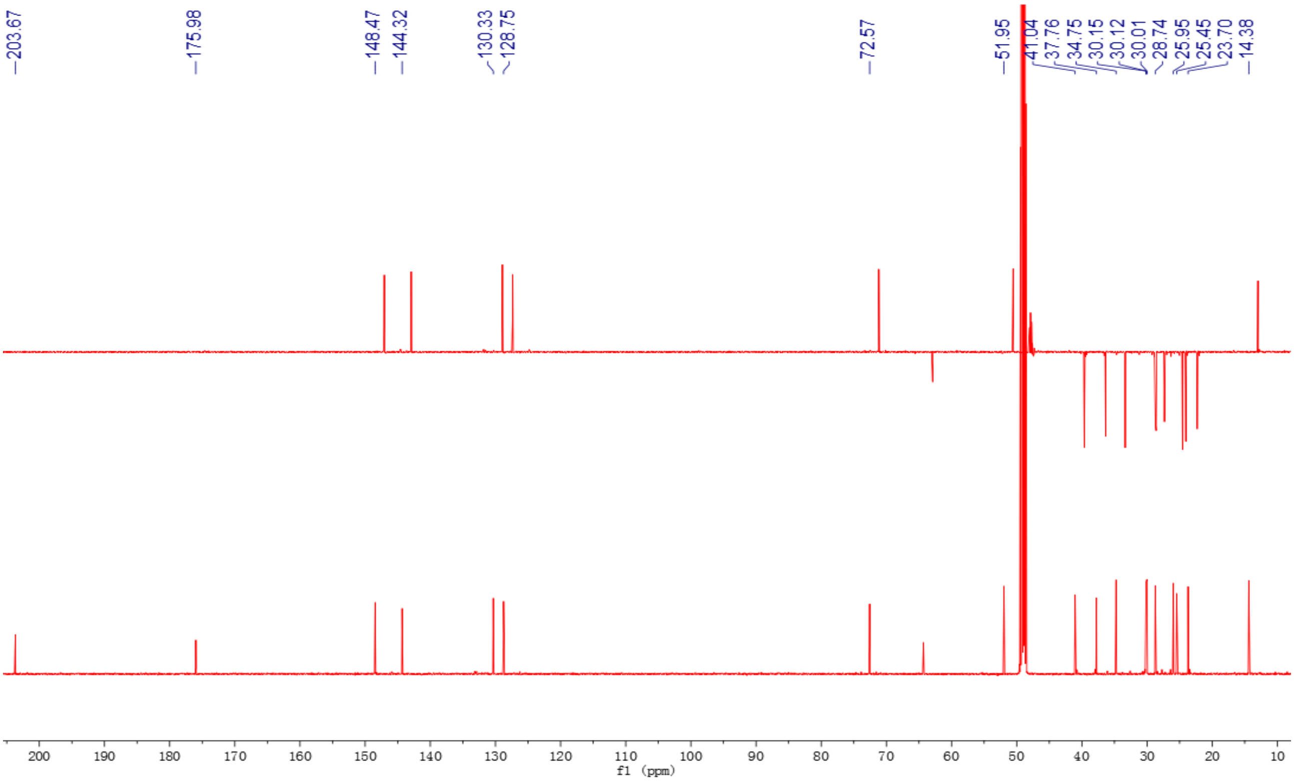

Figure S5. The ^{1}H-^{1}H COSY spectrum of **1**/**2** in CD_3OD.

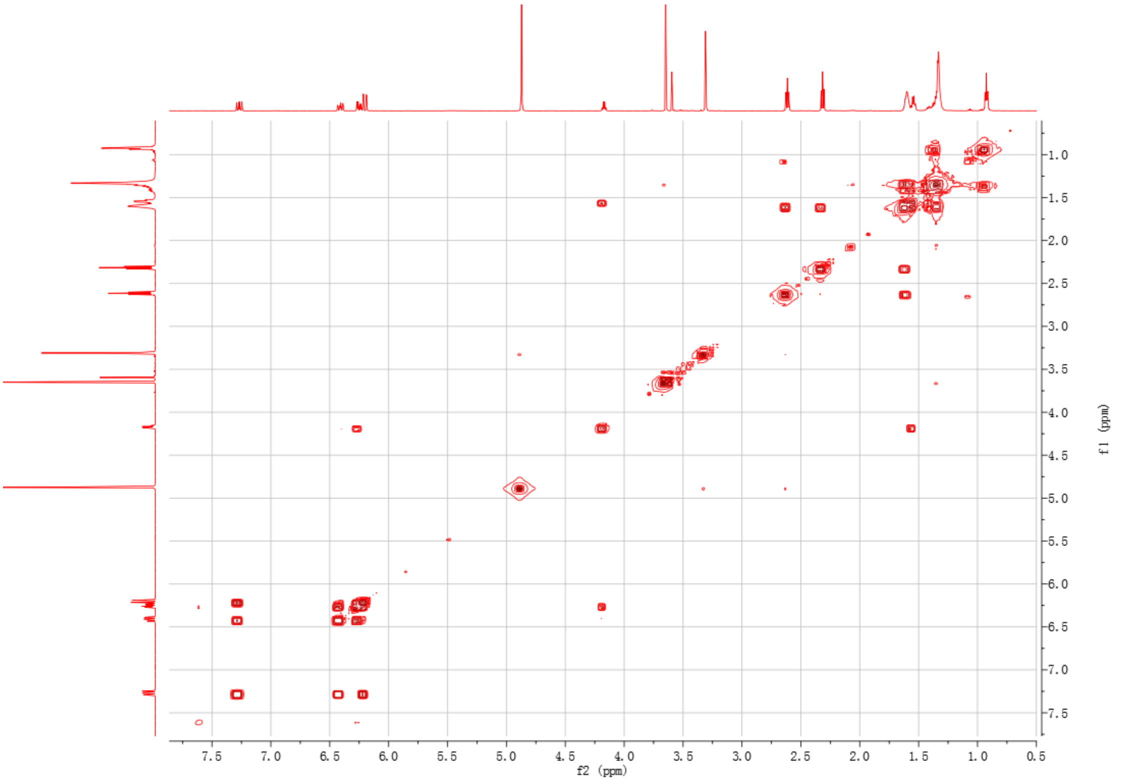

Figure S6. The HSQC spectrum of **1**/**2** in CD_3OD.

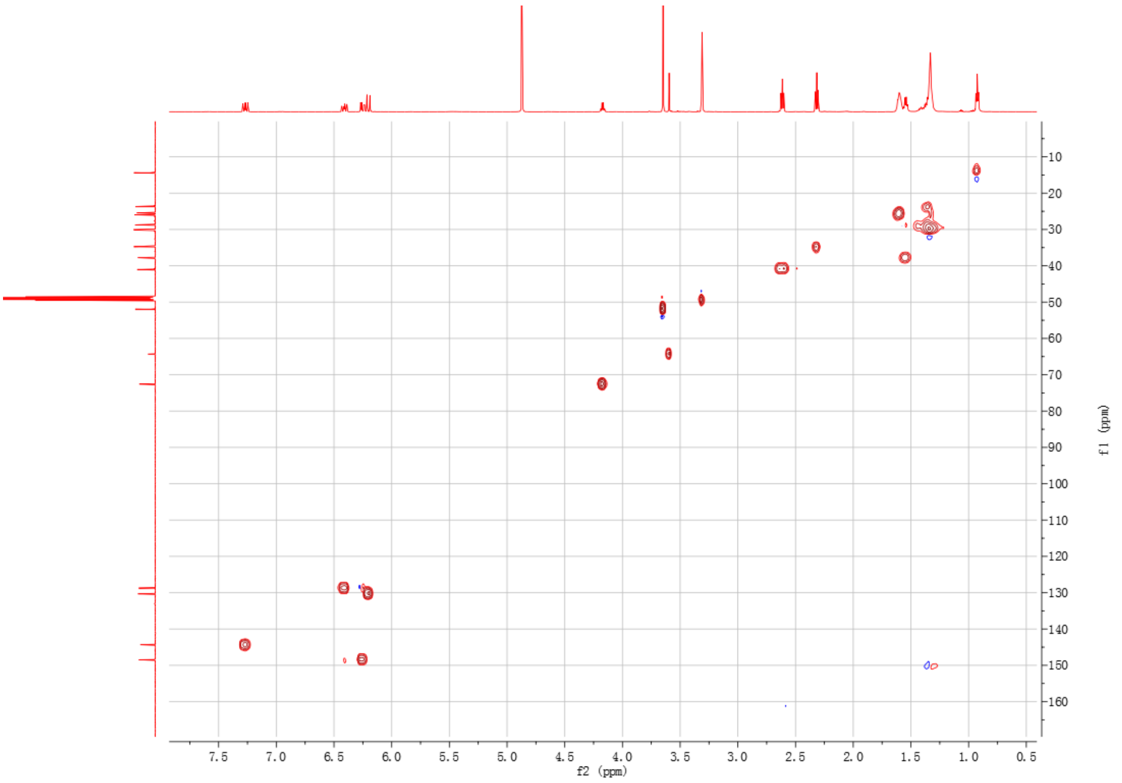

Figure S7. The HMBC spectrum of **1**/**2** in CD_3OD.

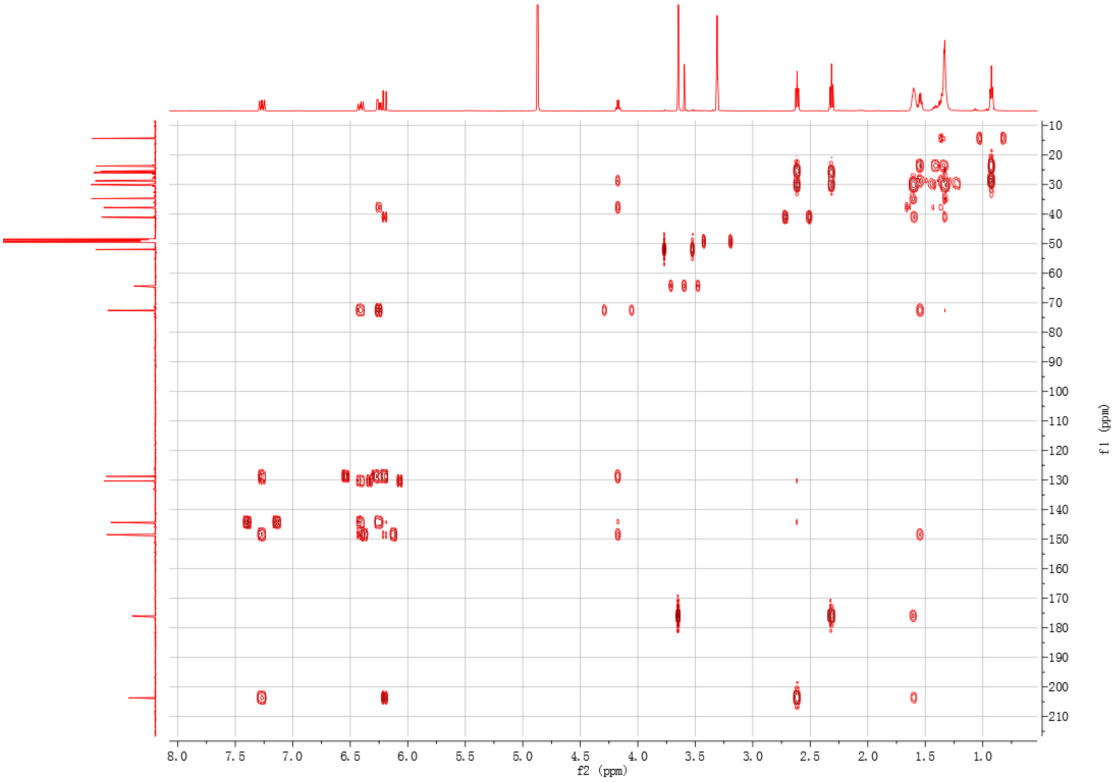

Figure S8. The (+)-HRESIMS spectrum of **1**/**2**.

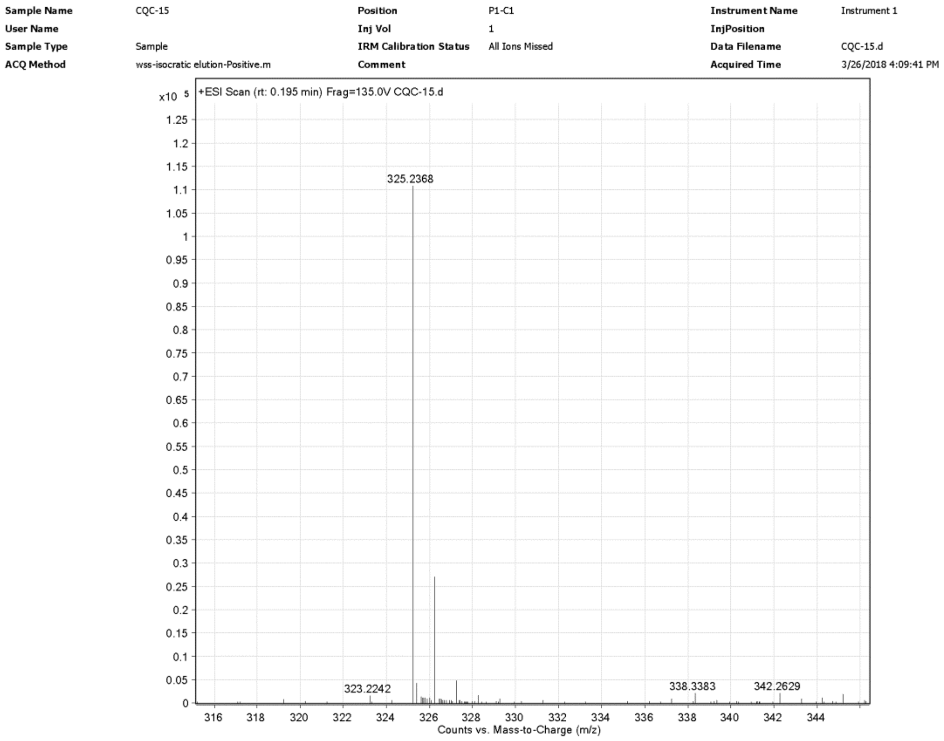

Figure S9. The ^{1}H NMR spectrum of **3**/**4** in CD_3OD.

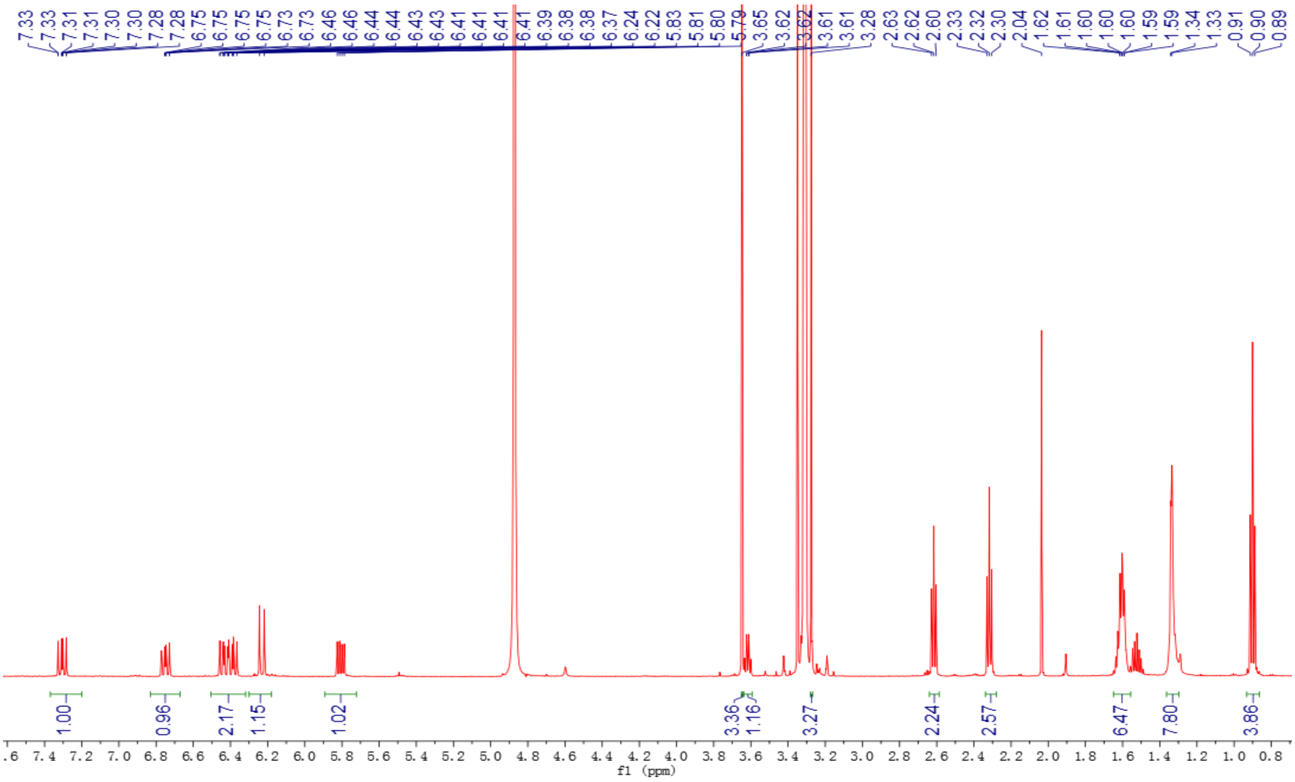

Figure S10. The ^{13}C NMR spectrum of **3**/**4** in CD_3OD.

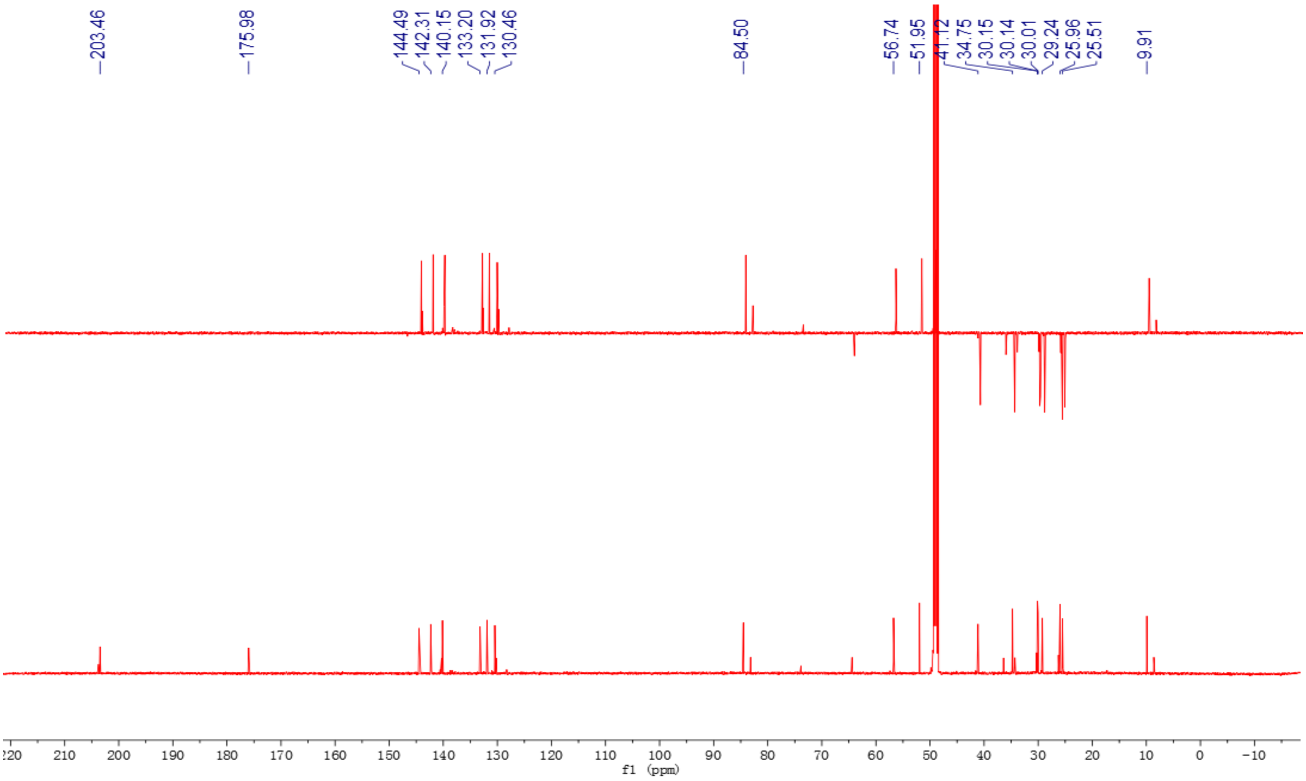

Figure S11. The ^{1}H-^{1}H COSY spectrum of **3**/**4** in CD_3OD.

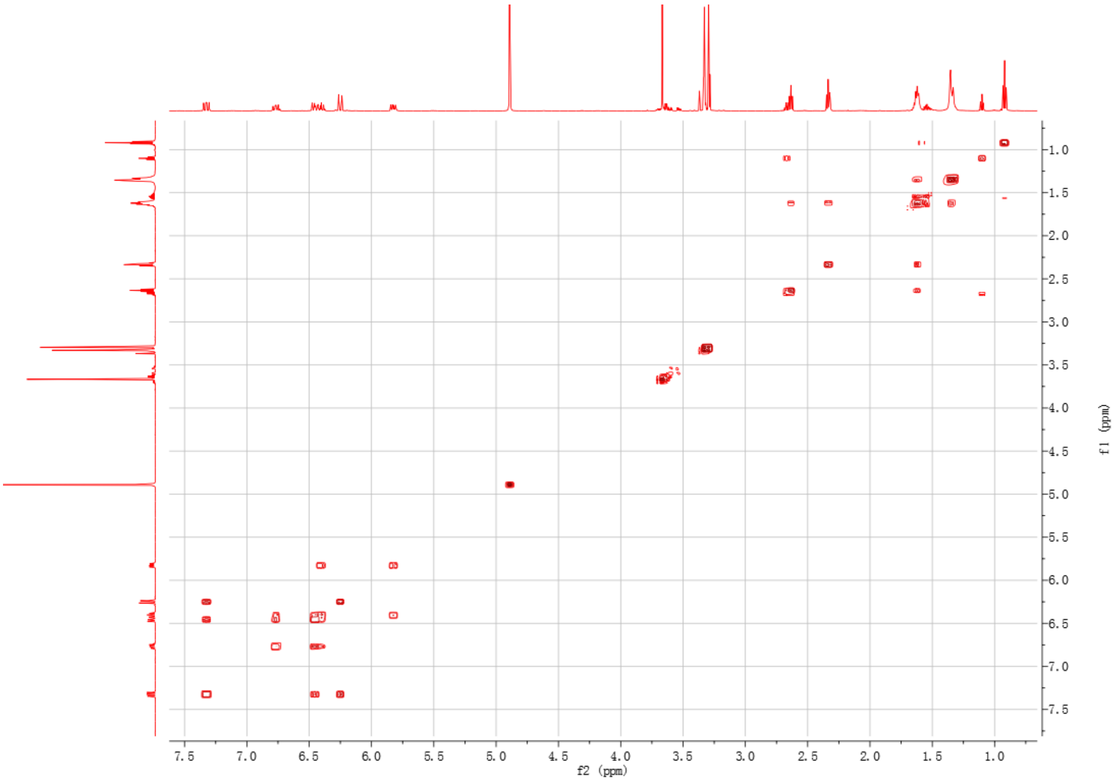

Figure S12. The HSQC spectrum of **3**/**4** in CD_3OD.

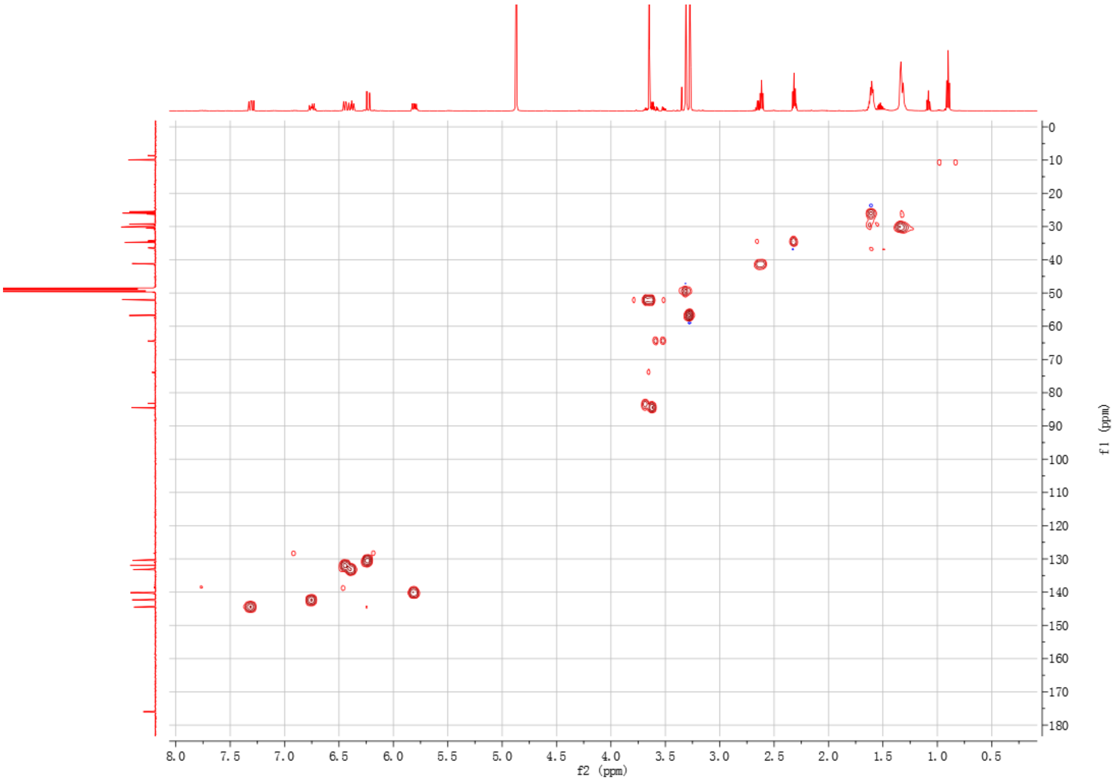

Figure S13. The HMBC spectrum of **3**/**4** in CD_3OD.

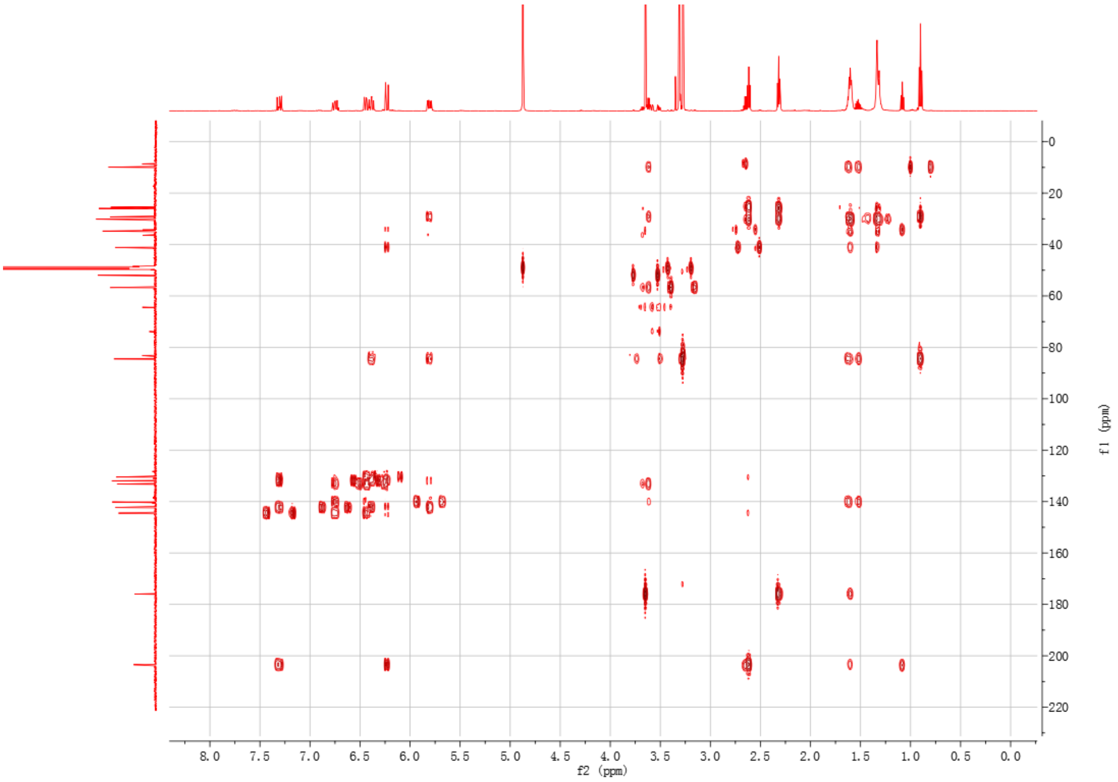

Figure S14. The (+)-HRESIMS spectrum of **3**/**4**.

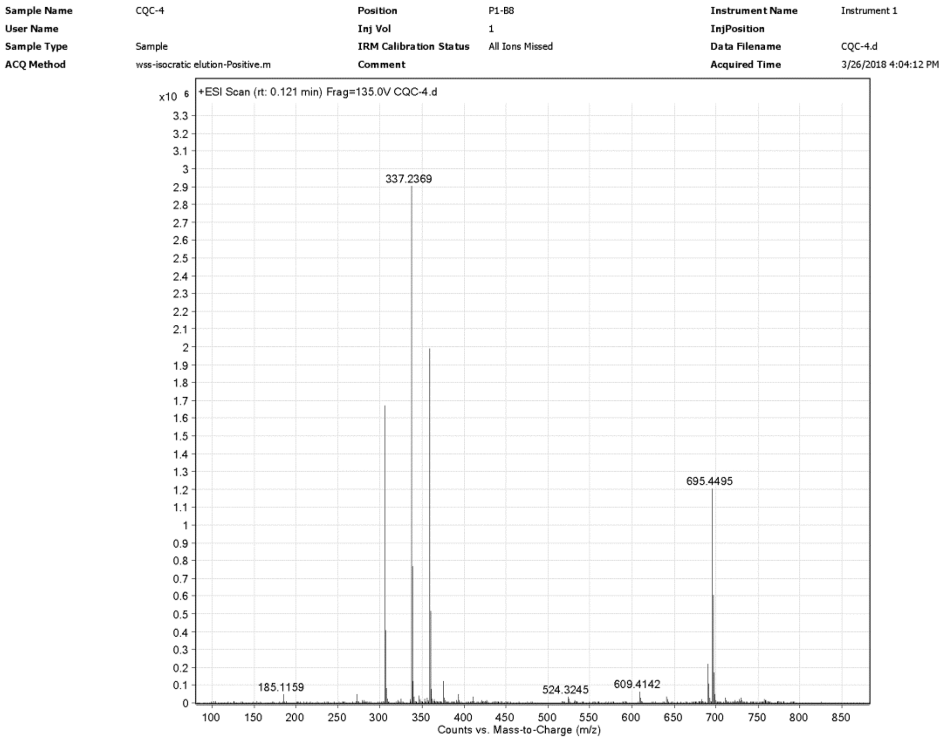

Figure S15. The ^{1}H NMR spectrum of **5**/**6** in $CDCl_3$.

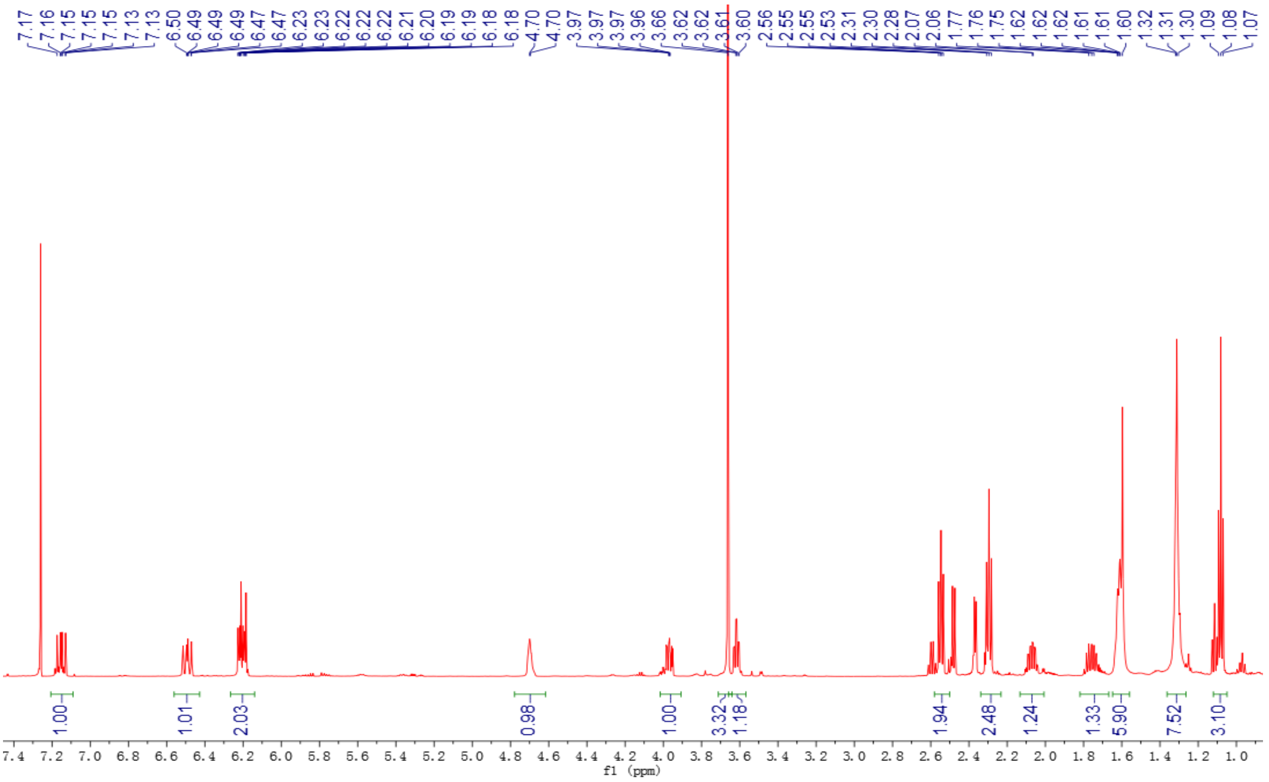

Figure S16. The ^{13}C NMR spectrum of **5**/**6** in $CDCl_3$.

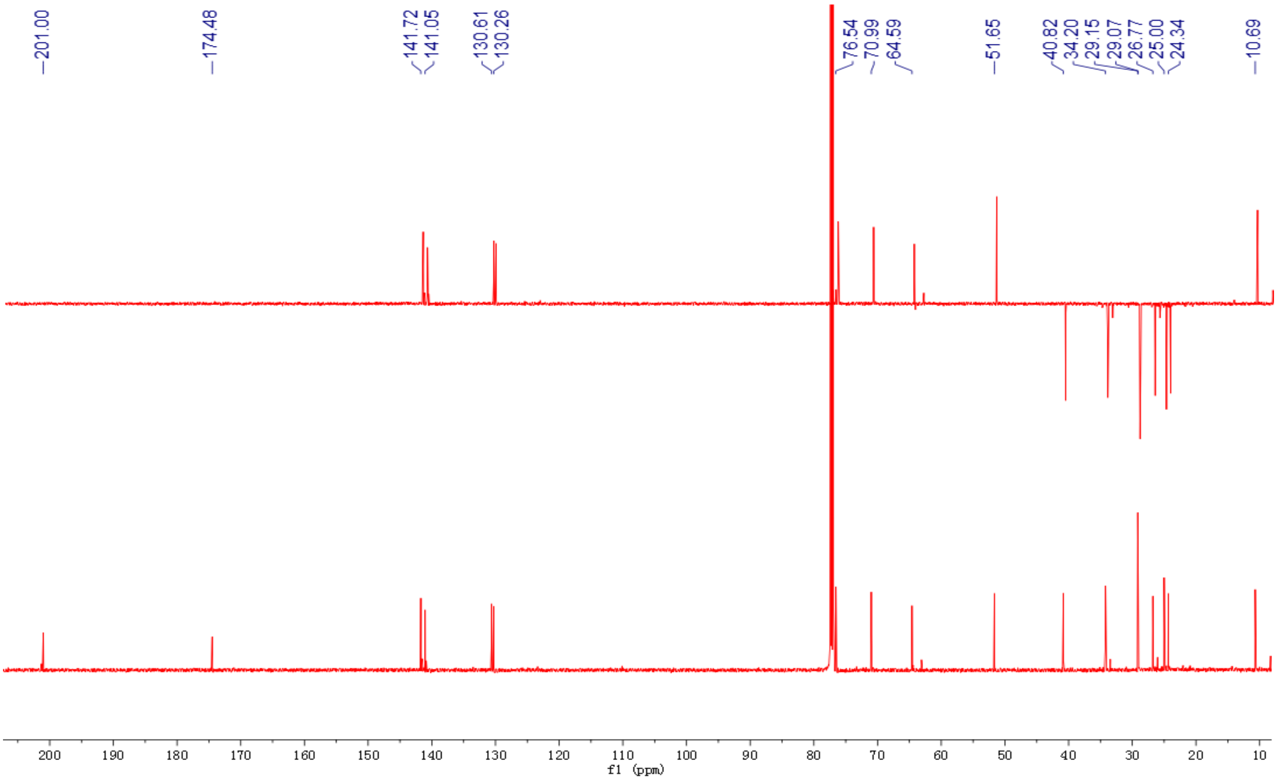

Figure S17. The ^{1}H-^{1}H COSY spectrum of **5**/**6** in $CDCl_3$.

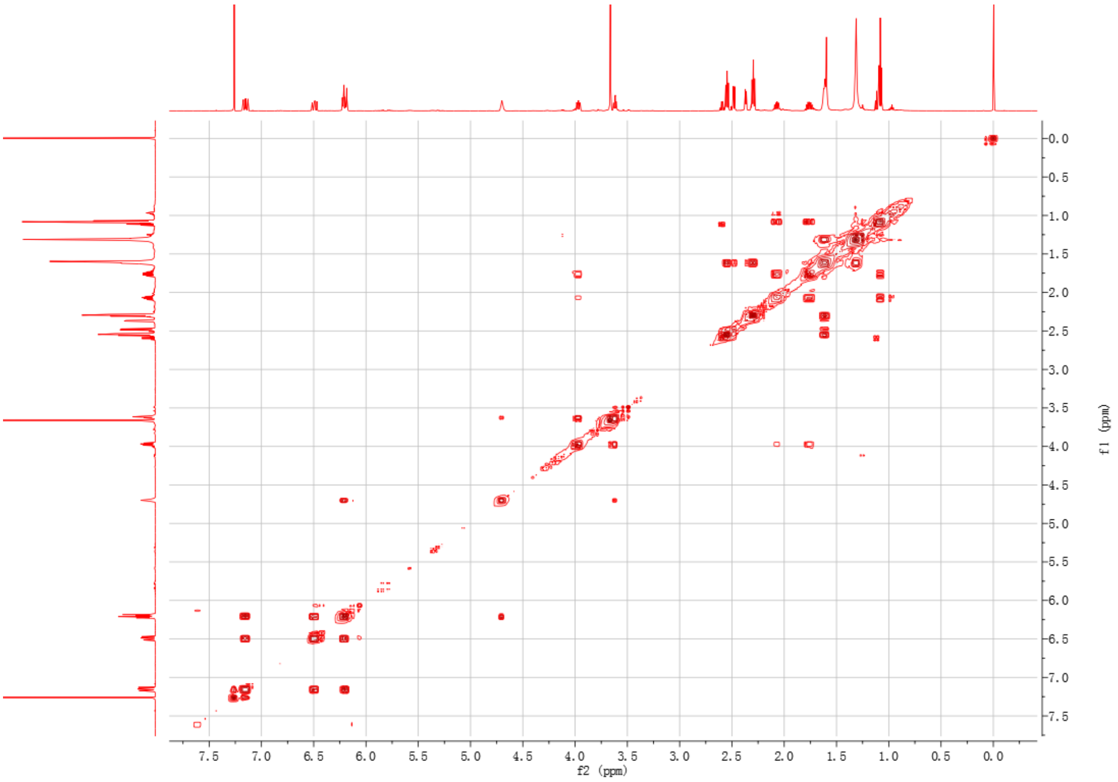

Figure S18. The HSQC spectrum of **5**/**6** in $CDCl_3$.

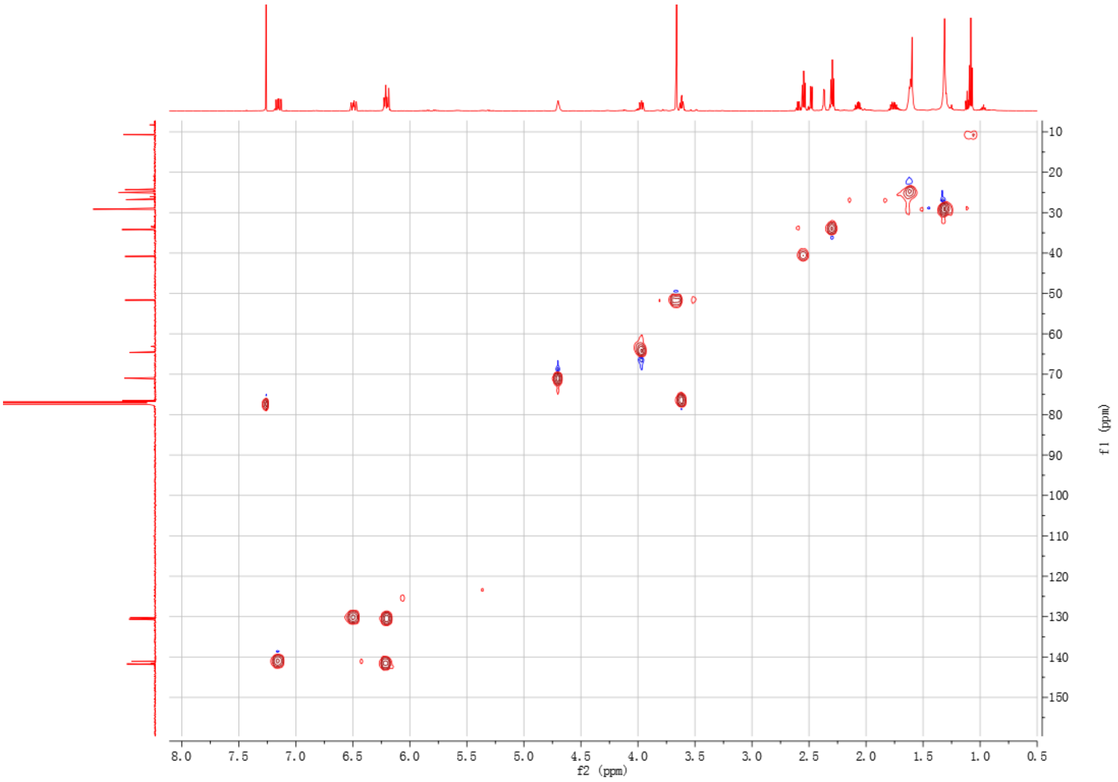

Figure S19. The HMBC spectrum of **5**/**6** in $CDCl_3$.

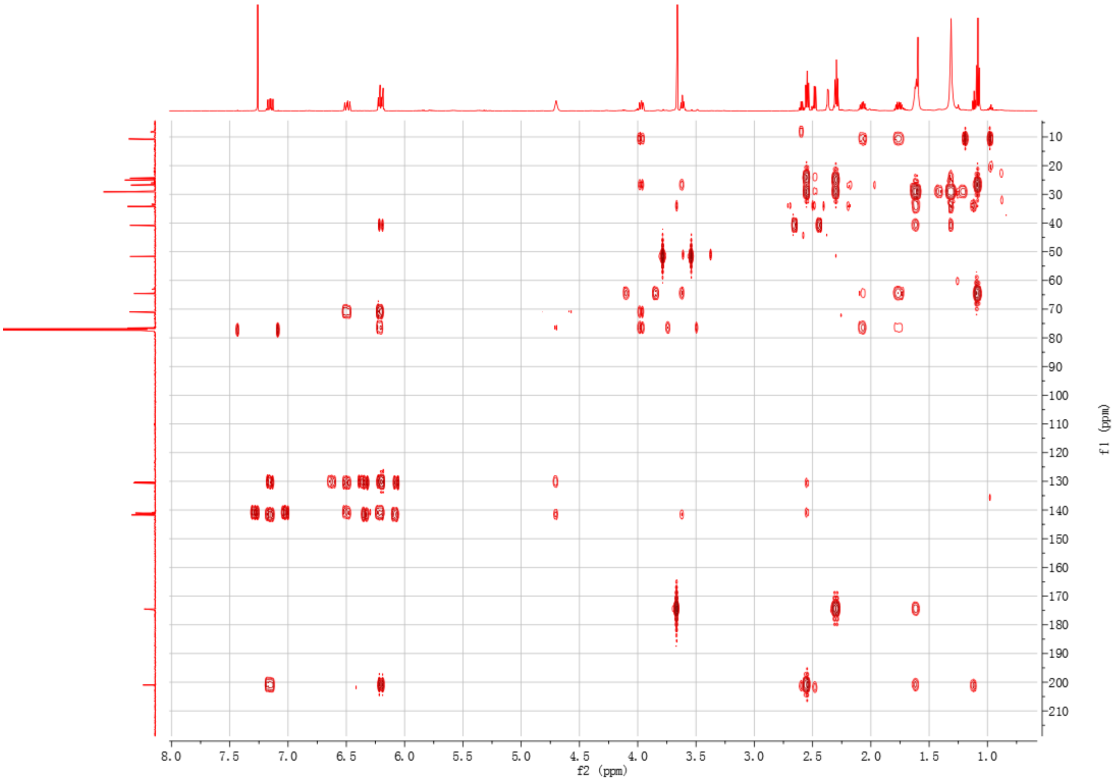

Figure S20. The (+)-HRESIMS spectrum of **5**/**6**.

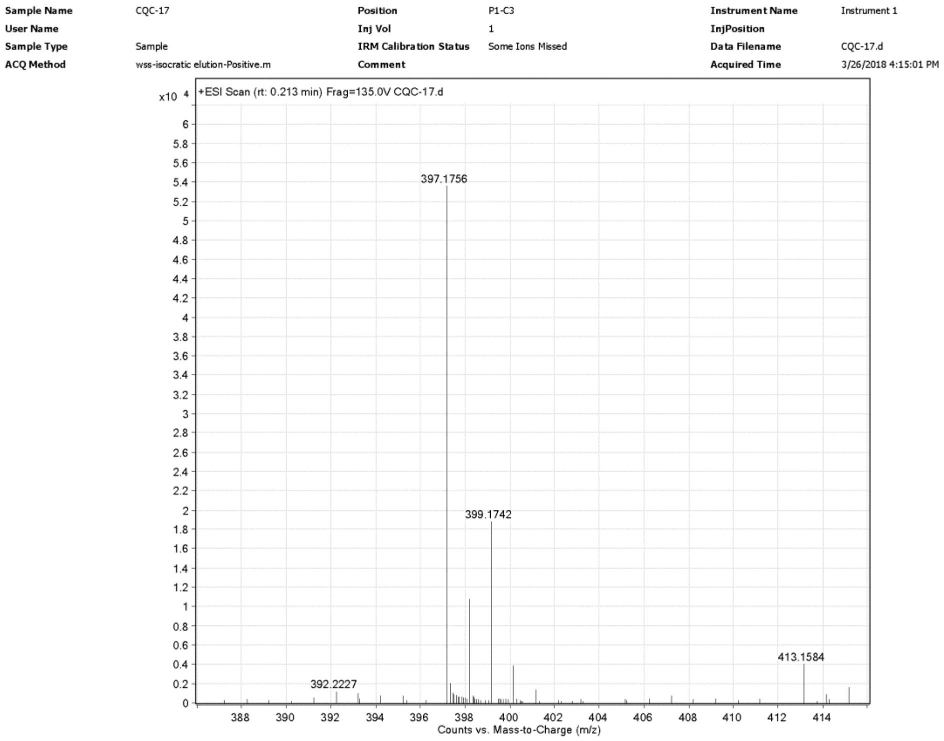

Figure S21. The ^{1}H NMR spectrum of **9** in $CDCl_3$.

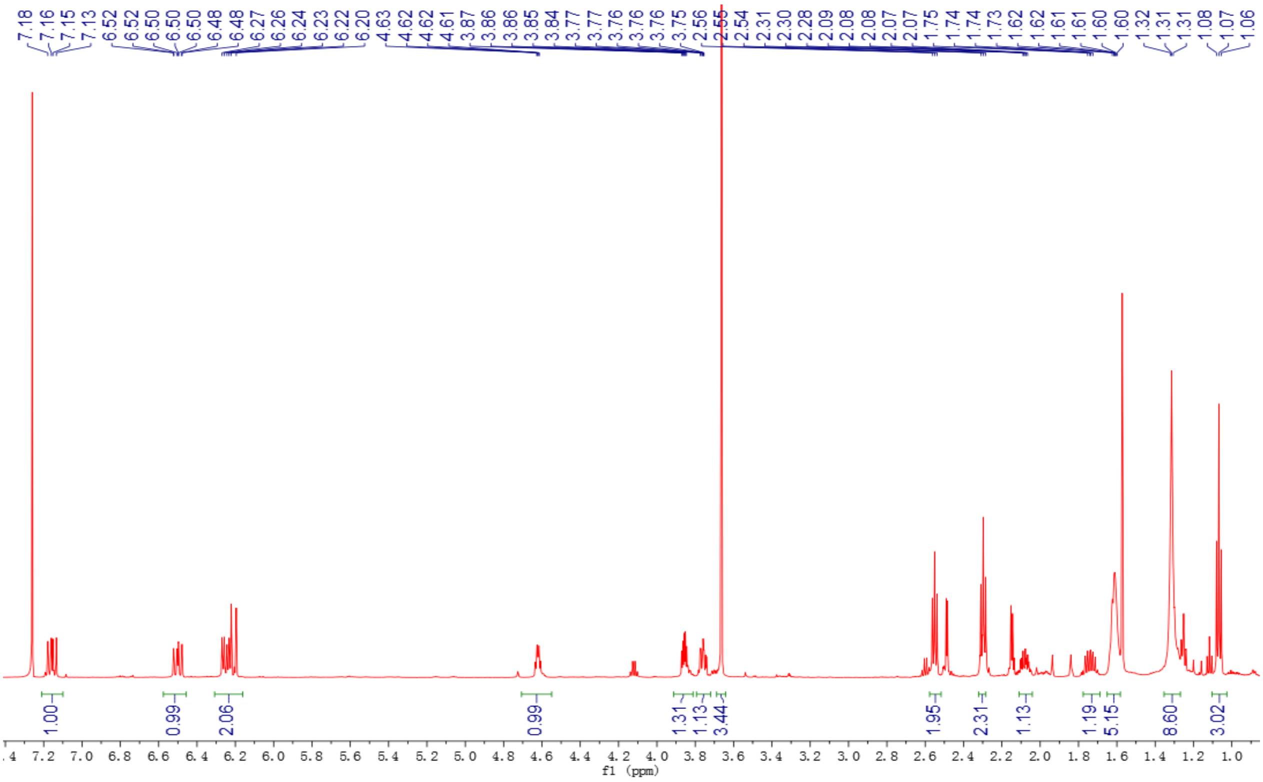

Figure S22. The ^{13}C NMR spectrum of **9** in $CDCl_3$.

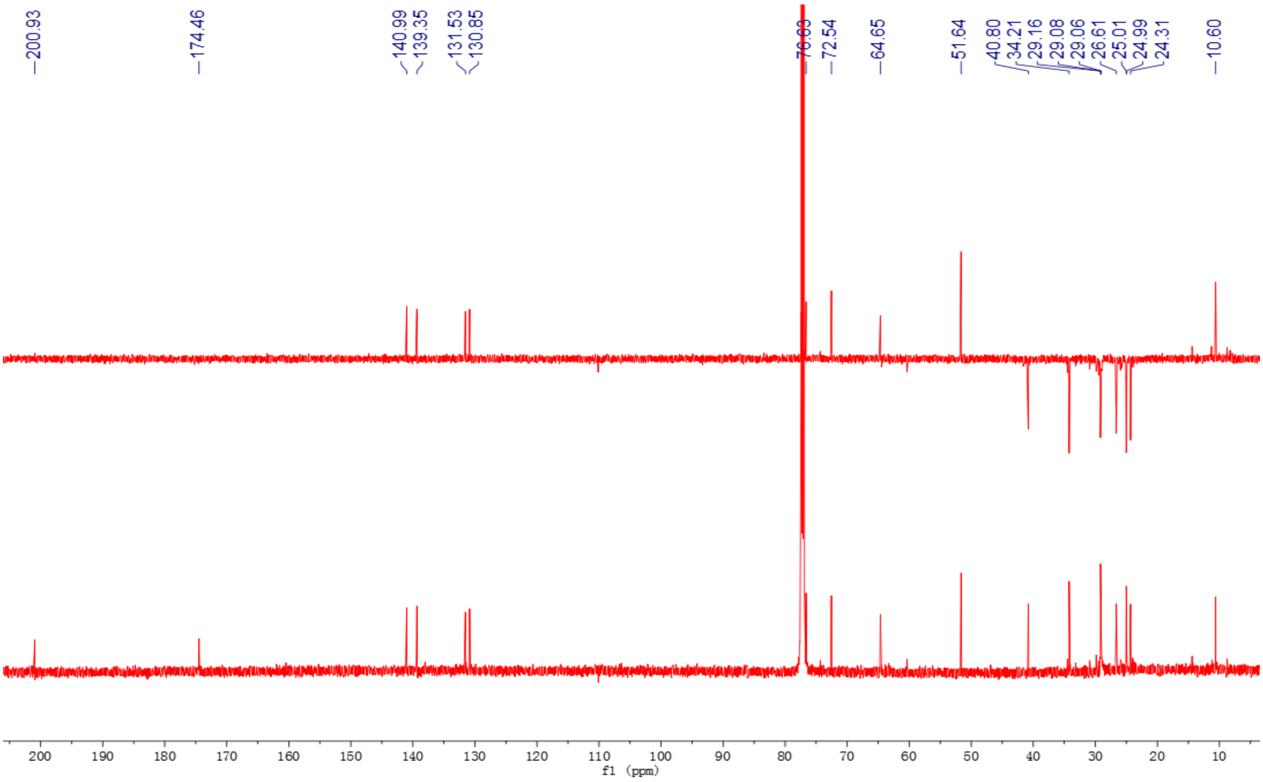

Figure S23. The ^1H-^1H COSY spectrum of **9** in $CDCl_3$.

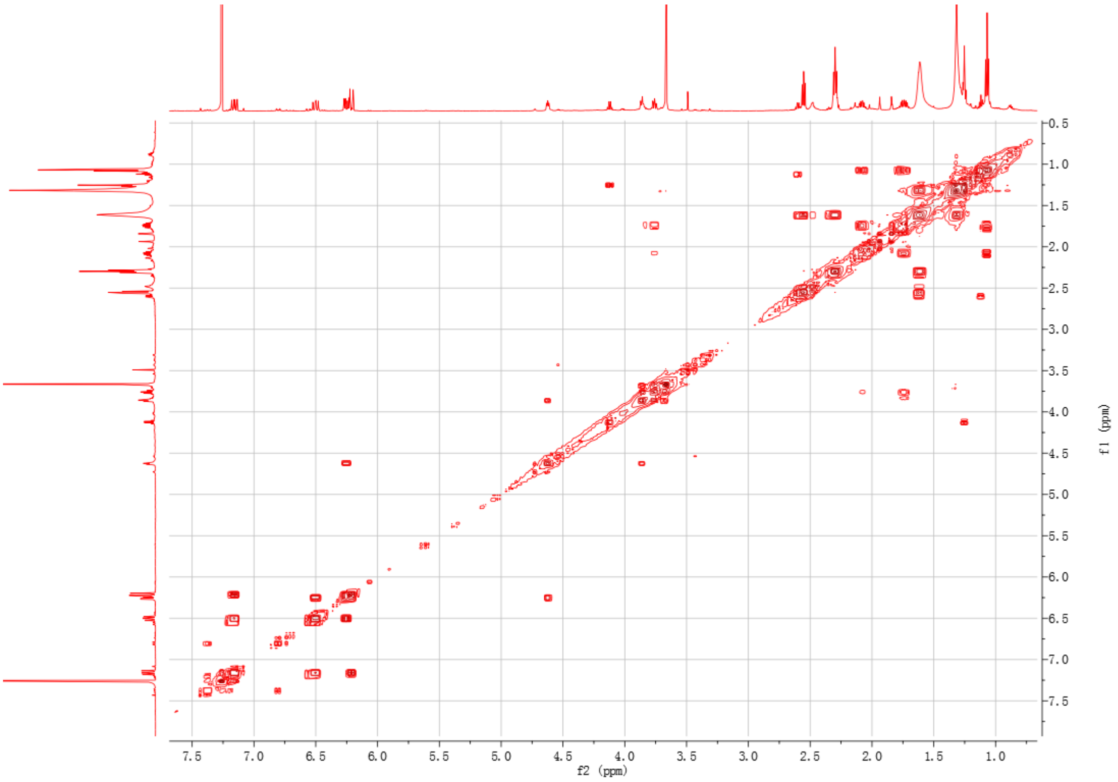

Figure S24. The HSQC spectrum of **9** in $CDCl_3$.

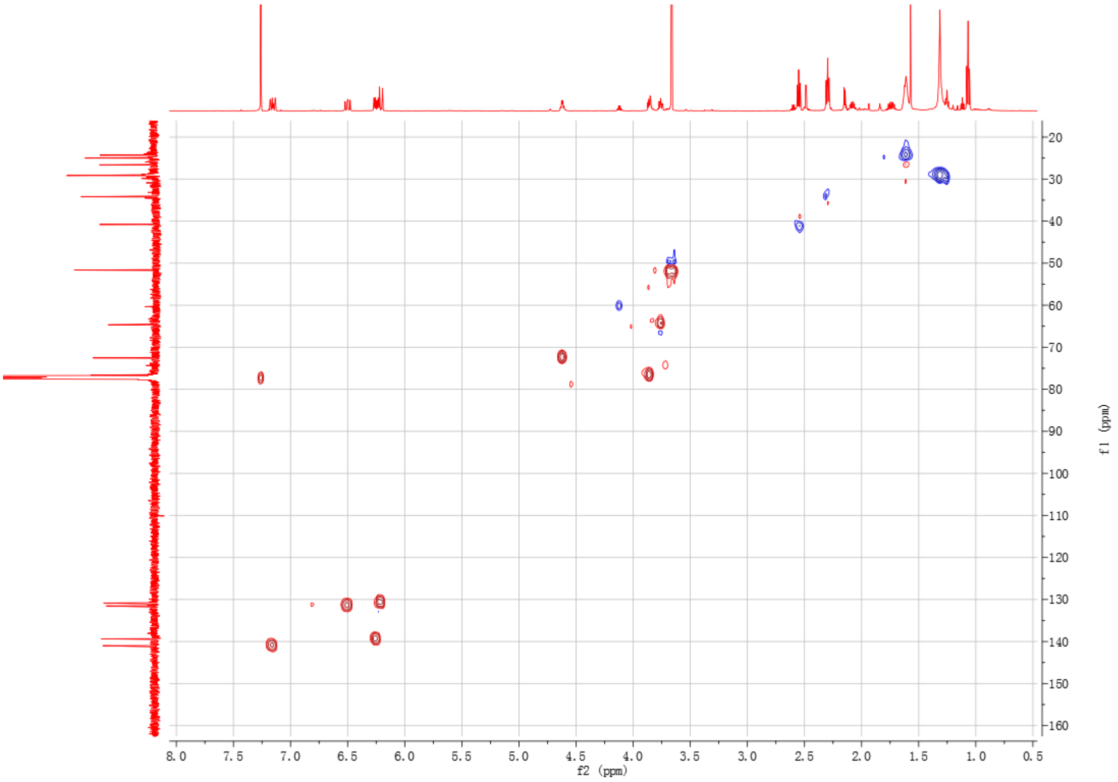

Figure S25. The HMBC spectrum of **9** in $CDCl_3$.

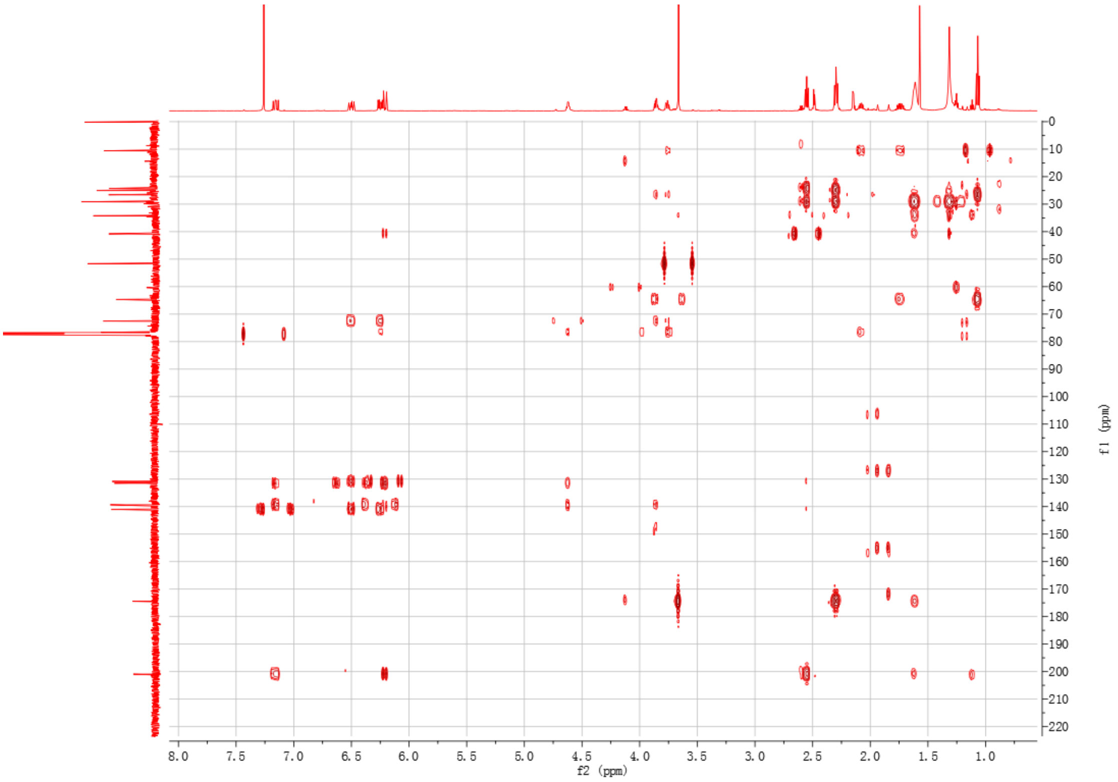

Figure S26. The (+)-HRESIMS spectrum of **9**.

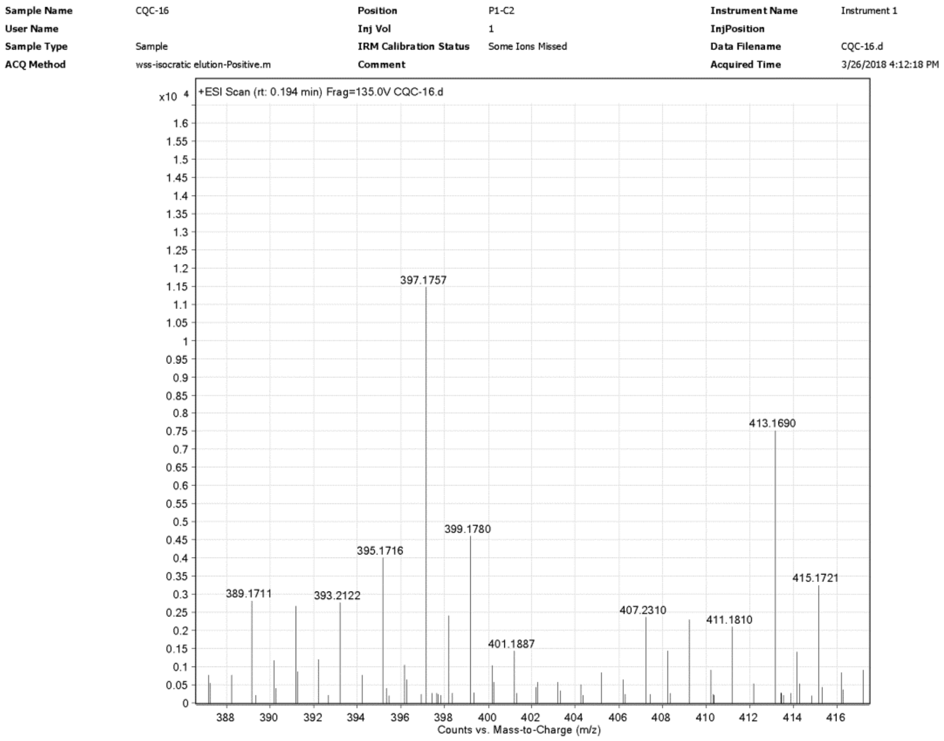

Table S1. Preliminary antimicrobial assay results (tested at 50 μM).

No.	EC	SA	BS	PA	CA
**1**	8.47%	6.35%	-60.04%	0.18%	1.80%
**2**	11.15%	8.85%	-18.46%	-0.43%	7.12%
**7**	22.65%	10.66%	-41.62%	4.40%	10.62%
**8**	14.03%	8.25%	-44.04%	5.11%	-3.48%
**10**	7.36%	9.23%	-61.78%	0.89%	12.04%
**11**	14.19%	7.05%	-19.20%	9.02%	16.79%
**12**	29.48%	13.77%	16.68%	2.29%	2.67%
**13**	7.86%	24.45%	-94.47%	5.44%	9.27%
**14**	9.57%	4.33%	-92.05%	8.68%	39.38%
**15**	-3.54%	8.75%	-51.87%	9.32%	43.54%
**17**	-1.14%	6.19%	-69.79%	2.61%	-13.91%
**18**	11.07%	21.41%	-109.43%	6.82%	12.21%

EC: *Escherichia coli* ATCC 8739; SA: *Staphylococcus aureus* ATCC 25923; BS: *Bacillus subtilis* ATCC 6633 ; PA: *Pseudomonas aeruginosa* ATCC 9027; CA: *Candida albicans* ATCC 10231.

Table S2. Preliminary anti-acetylcholinesterase and anti-inflammatory assay results.

No.	anti-acetylcholinesterase[a]	anti-inflammatory[b] (NO inhibition)	anti-inflammatory[b] (Cell viability)
**1**	23.19%	38.91%	-
**2**	0%	45.60%	-
**3**	14.27%	15.83%	-
**4**	14.32%	-9.72%	-
**5**	1.44%	27.61%	-
**6**	3.79%	15.14%	-
**7**	0%	22.70%	-
**8**	0%	23.13%	-
**10**	18.67%	31.42%	-
**11**	21.62%	30.41%	-
**12**	13.83%	-2.68%	-
**13**	3.21%	71.12%	105.2%
**14**	6.06%	39.31%	-
**15**	12.64%	28.74%	-
**16**	0%	23.63%	-
**17**	0%	45.51%	-
**18**	9.26%	60.21%	119.8%
**19**	0%	45.93%	-

[a] Tested at 50 μM; [b] Tested at 20 μM.
